# Structure of Schlafen13 reveals a new class of tRNA/rRNA- targeting RNase engaged in translational control

**DOI:** 10.1038/s41467-018-03544-x

**Published:** 2018-03-21

**Authors:** Jin-Yu Yang, Xiang-Yu Deng, Yi-Sheng Li, Xian-Cai Ma, Jian-Xiong Feng, Bing Yu, Yang Chen, Yi-Ling Luo, Xi Wang, Mei-Ling Chen, Zhi-Xin Fang, Fu-Xiang Zheng, Yi-Ping Li, Qian Zhong, Tie-Bang Kang, Li-Bing Song, Rui-Hua Xu, Mu-Sheng Zeng, Wei Chen, Hui Zhang, Wei Xie, Song Gao

**Affiliations:** 1 State Key Laboratory of Oncology in South China, Collaborative Innovation Center for Cancer Medicine, Sun Yat-Sen University Cancer Center, Guangzhou, 510060 Guangdong China; 2 State Key Laboratory for Biocontrol, School of Life Sciences, Sun Yat-Sen University, Guangzhou, 510060 Guangdong China; 3 Laboratory for Functional Genomics and Systems Biology, Berlin Institute for Medical Systems Biology, Berlin, 13125 Germany; 4 Department of Biology, Southern University of Science and Technology, Shenzhen, 518055 Guangdong China; 5 Key Laboratory of Tropical Disease Control of Ministry of Education, Institute of Human Virology, Zhongshan School of Medicine, Sun Yat-Sen University, Guangzhou, 510080 Guangdong China; 6 Medi-X Institute, SUSTech Academy for Advanced Interdisciplinary Studies, Southern University of Science and Technology, Shenzhen, 518055 Guangdong China; 7 Present Address: Laboratory of Metabolism, Center for Cancer Research, National Cancer Institute, National Institute of Health, Bethesda, MD 20892 USA; 8 Present Address: Division of Theoretical Systems Biology, German Cancer Research Center, Heidelberg, 69120 Germany

## Abstract

Cleavage of transfer (t)RNA and ribosomal (r)RNA are critical and conserved steps of translational control for cells to overcome varied environmental stresses. However, enzymes that are responsible for this event have not been fully identified in high eukaryotes. Here, we report a mammalian tRNA/rRNA-targeting endoribonuclease: SLFN13, a member of the Schlafen family. Structural study reveals a unique pseudo-dimeric U-pillow-shaped architecture of the SLFN13 N′-domain that may clamp base-paired RNAs. SLFN13 is able to digest tRNAs and rRNAs in vitro, and the endonucleolytic cleavage dissevers 11 nucleotides from the 3′-terminus of tRNA at the acceptor stem. The cytoplasmically localised SLFN13 inhibits protein synthesis in 293T cells. Moreover, SLFN13 restricts HIV replication in a nucleolytic activity-dependent manner. According to these observations, we term SLFN13 RNase S13. Our study provides insights into the modulation of translational machinery in high eukaryotes, and sheds light on the functional mechanisms of the Schlafen family.

## Abstract (web summary)

Translation inhibition is a strategy for organisms to overcome various environmental stresses including viral infections. Here the authors show that a tRNA/rRNA-targeting RNase Schlafen13 inhibits protein synthesis by directly digesting cytoplasmic tRNA and rRNA with the ability to restrict viral propagation.

## Introduction

Modulation of translational machinery is a critical strategy for cells to overcome varied environmental assaults including viral infections^1–4^. Known mechanisms involve the intervention with eukaryotic translation initiation factor 4F complex (eIF4F complex) or eIF2–GDP, which promotes the formation of so-called stress granules and eventually causes rapid inhibition of bulk protein synthesis^1,5^. Besides the regulation of translation initiation, the turnover of tRNA and rRNA has also been suggested to play an important role in translational control^6–8^. Stress-induced cleavage of tRNA and rRNA in the cytosol is observed in both prokaryotes and eukaryotes, which causes protein synthesis arrest and results in growth inhibition or eventually, cell death^8–10^. In addition, emerging evidence suggests that the cleavage products of tRNA and rRNA may act as siRNA or miRNA in promoting translation inhibition^11–13^. Up to now, few tRNases involved in this process are known in high eukaryotes. These enzymes, such as human angiogenin, are normally secreted or sequestered proteins that gain access to cytosolic tRNAs upon cellular stress^8,14,15^. More RNases responsible for stress-induced tRNA/rRNA turnover are yet to be identified.

Schlafen proteins (SLFNs) were first discovered in regulating mice thymocyte development^16^, and then found to span across mammals with great diversity^17^. There are ten known or predicted SLFNs in mice which can be categorised into three subgroups by size, whereas only five SLFNs have been identified in humans, and four of them (SLFN5, SLFN11, SLFN13 and SLFN14) belong to the largest subgroup III^17^ (Supplementary Fig. 1a). SLFNs were reported to be interferon (IFN)-inducible^18–21^, and their functions have been implicated in various biological processes including cell differentiation, tumour suppression, immune response regulation and the restriction of viruses^22^. Specifically, human SLFN11 has been reported to bind tRNAs and restrict HIV protein synthesis possibly by means of codon-bias discrimination^23^. However, the detailed structural properties and functional mechanisms of SLFNs still remain unclear.

All SLFNs share a specific N-terminal AAA_4 domain (Pfam04326, termed N′-domain in this paper) which has a very limited sequence homology to other known proteins^17^ and was reportedly involved in tRNA manipulation for certain members^23,24^. In addition, a SWADL domain is present in the members of subgroups II and III, and the largest SLFN subgroup is characterised by a C-terminal region that is homologous to the superfamily I of RNA helicases^25^ (Supplementary Fig. 1a). To gain a functional insight into the Schlafen family, we determined the crystal structure of SLFN13′s N′-domain. Investigation of the structure and subsequent functional analysis reveals that SLFN13 is a novel tRNA/rRNA-targeting RNase with potent anti-HIV activity. These results expand our vision of translational control in high eukaryotes and functional mechanisms of the Schlafen family.

## Results

### Overall structure of the SLFN13 N′-domain

**Fig. 1:**
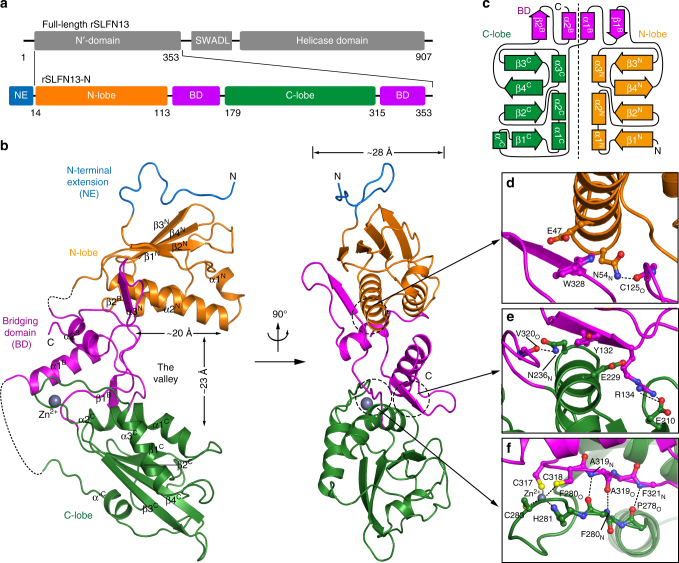
Structure of rSLFN13 N′-domain. **a** Schematic representation showing the organisation of crystallised rSLFN13_14–353_ based on full-length rSLFN13. NE denotes the N-terminal extension coded by the DNA sequence from the vector; BD denotes bridging domain. Elements for rSLFN13_14–353_ are assigned according to the structure. Borders of each element are indicated by residue numbers. **b** Structure of rSLFN13_14–353_. Domains of rSLFN13_14–353_ are indicated and coloured as in **a**. Disordered loops are shown as dashed lines. The Zn^2+^ ion in the zinc finger is shown as a grey sphere. α-helices and β-strands of each domain are specified. The size of the valley is indicated. **c** The topology diagram of rSLFN13_14–353_. Secondary structural elements were not drawn to scale. Elements of rSLFN13_14–353_ are named and coloured as in **a**. A dashed line is drawn to indicate the pseudo-symmetry of rSLFN13_14–353_. **d** Interactions between BD and N-lobe. Side chains of involved residues are shown in the same colour as the domains they belong to. **e** Interactions between BD and C-lobe. Note the similarity with **d**. **f** Extra interactions between BD and C-lobe. Note the CCCH-type zinc finger

The structure of rat (r)SLFN13 (related to human [h]SLFN13 and mouse [m]SLFN8) N′-domain containing residues 14–353 was solved at 3.18 Å, where the anomalous signals of 31 selenium atoms in the asymmetric unit were used for phasing. In the calculated electron density, the main chains and most side chains were clearly traceable, enabling us to unambiguously assign them. The final model was refined to an *R*_free_ of 0.247 (Table 1, Fig. 1a, b; Supplementary Fig. 1b). 15 vector-encoded residues fused N-terminally to rSLFN13_14–353_ are clearly discernable in the model. The U-pillow-shaped rSLFN13_14–353_ is composed of an N-terminal lobe (N-lobe), a C-terminal lobe (C-lobe) and a bridging domain (BD), which together create a huge valley with a width of ~23 Å and a depth of ~20 Å. rSLFN13_14–353_ exhibits a pseudo-dimeric organisation: each lobe contains a four-stranded β-sheet surrounded by three α-helices, and the bipartite BD derived from two separate regions of the primary sequence also bears a two-fold symmetry in topology (Fig. 1b, c; Supplementary Fig. 1c).

**Table 1:** Crystallographic data collection and refinement statistics

	rSLFN13_14–353_	rSLFN13_14–353_
*Data collection*		
Data set	Native	SeMet derivative
Space group	C2	C2
*Cell dimensions*		
*a*, *b*, *c* (Å)	172.05, 134.33, 77.66	171.96, 134.65, 77.69
*α*, *β*, *γ* (°)	90, 105.51, 90	90, 104.72, 90
Wavelength (Å)	0.97853	0.97931
Resolution (Å)	50.0–3.18 (3.38–3.18)^a^	50.0–3.50 (3.71–3.50)
*R*_sym_	0.049 (0.544)	0.086 (0.525)
*I/σI*	17.08 (2.05)	12.00 (2.81)
Completeness (%)	99.2 (98.4)	99.4 (99.3)
Redundancy	3.41 (3.36)	3.84 (3.87)
*Refinement*		
Resolution (Å)	35.24–3.18 (3.30–3.18)	
No. reflections	28,212 (2660)	
*R*_work_/*R*_free_	0.200/0.247	
Average *B*-factor	108.67	
*R.m.s. deviations*		
Bond lengths (Å)	0.003	
Bond angles (°)	0.635	
*Ramachandran plot (%)*		
Favoured	94.48	
Allowed	4.97	
Outliers	0.55	

^a^ Values in parentheses are for highest-resolution shell

The α2^N^ and α2^C^ of the two lobes individually wedge into the grooves of the butterfly-like BD at both sides (Fig. 1b). N-lobe-BD association includes a hydrogen bond between Asn54 of α2^N^ and the backbone oxygen of Cys125, as well as a carboxylate-π-stacking between Glu47 of α2^N^ and Trp328 of β2^Β^ (Fig. 1d). Corresponding associations were found at equivalent positions of C-lobe-BD interface (Asn236-Val320_O_ and Glu229-Tyr132), although an extra salt bridge between Glu210 and Arg134 is also present (Fig. 1e). Moreover, a bulging loop of C-lobe is tightly associated with BD by a line of main chain hydrogen bonds and a CCCH-type zinc finger (Fig. 1f; Supplementary. 1d). Mutation on any of the Zn^2+^-coordinating residue rendered rSLFN13_1–353_ insoluble, suggesting that the zinc finger is crucial for the folding of rSLFN13. rSLFN13_1–353_ constructs were eluted as monomers in the right-angle light scattering (RALS) analysis, whereas the crystallised His_6_-tagged rSLFN13_14–353_ was a dimer (Supplementary Fig. 2a). The dimerisation of His_6_-tagged rSLFN13_14–353_ is likely mediated by the artificial N-terminus (Supplementary Fig. 2b). In agreement with this, His_6_-tagged rSLFN13_14–353_ showed much higher resistance to the proteolytic treatment targeting His_6_-tag compared to His_6_-tagged rSLFN13_1–353_ (Supplementary Fig. 2c). In this paper, the N′-domain constructs of SLFN13 are collectively termed SLFN13-N unless otherwise specified. The same nomenclature is also applied to other SLFNs.

### SLFN13 N′-domain cleaves tRNA in vitro

**Fig. 2:**
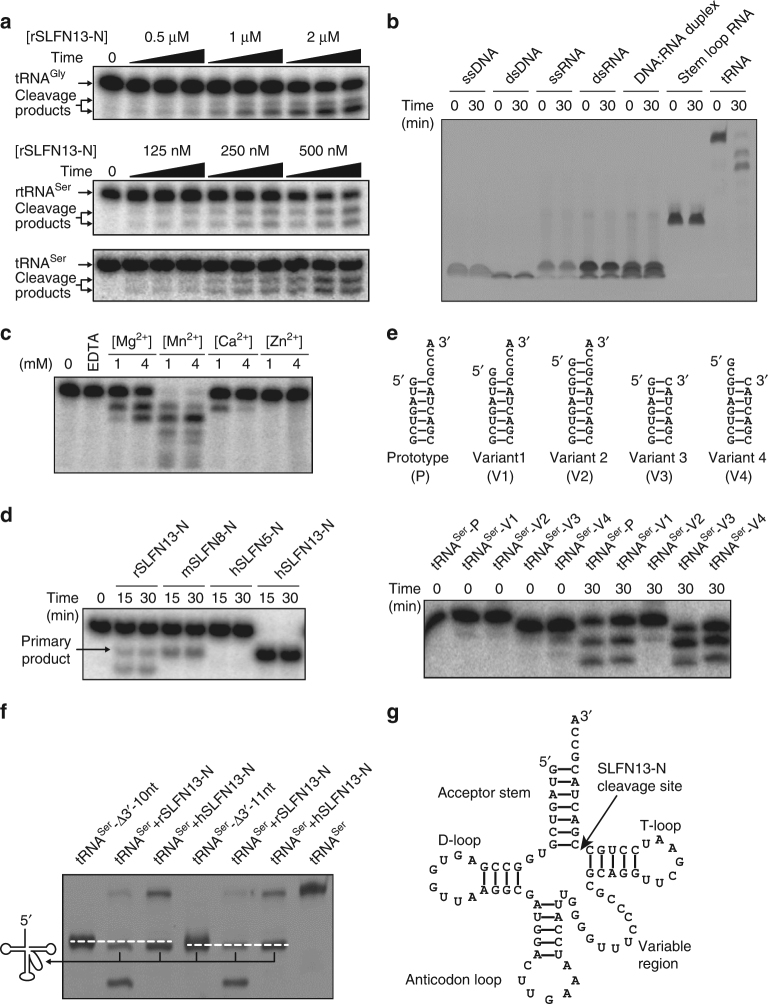
Endoribonuclease activity of SLFN13-N. **a** Dose-dependent and time-dependent tRNA digestion by rSLFN13-N in the presence of Mg^2+^. rSLFN13_1–353_ of indicated concentrations were incubated with [α-^32^P]-labelled human tRNA^Gly^, human tRNA^Ser^ or rat tRNA^Ser^ (rtRNA^Ser^) for 5, 15 and 30 min. **b** rSLFN13-N preferably cleaves tRNA. ssDNA/ssRNA single-stranded DNA/RNA, dsDNA/dsRNA double-stranded DNA/RNA. **c** Analysis of divalent cations as cofactor of rSLFN13-N for tRNA digestion. 500 nM rSLFN13_1–353_ was incubated with [α-^32^P]-labelled tRNA^Ser^ for 30 min. Four different divalent cations were individually tested. **d** Cleavage assay for different SLFN-N proteins. 250 nM rSLFN13_1–353_, mSLFN8_1–359_, hSLFN13_1–355_ or hSLFN5_12–334_ was individually incubated with [α-^32^P]-labelled tRNA^Ser^. Note that hSLFN13-N shows highest nucleolytic efficiency over human tRNA^Ser^. **e** Cleavage assay for rSLFN13-N on acceptor stem variants. Modifications of the variants are specified. 500 nM rSLFN13_1–353_ was used in each reaction. **f** tRNA cleavage pattern of SLFN13-N. The primary cleavage products from tRNA^Ser^ by rSLFN13_1–353_ and hSLFN13_1–355_ were compared with in vitro transcribed tRNA^Ser^ that lacks 3′-terminal 10 nt (tRNA^Ser^−3′-Δ10 nt) or 11 nt (tRNA^Ser^−3′-Δ11 nt), and portrayed to the left. The migration of two truncated tRNA^Ser^ markers is indicated by dashes lines. **g** Proposed primary cleavage site of SLFN13-N. The sequence of human tRNA^Ser^ is used here as representative

Close relatives of SLFN13 were reported to functionally associate with tRNA^23,24^, but the detailed mechanism has not been characterised yet. To understand whether and how SLFN13 is involved in tRNA manipulation, we tested the binding of SLFN13-N with in vitro transcribed human tRNA^Gly^ or tRNA^Ser^ (human tRNA transcripts were used in the functional assays throughout this paper, unless otherwise specified). tRNA^Gly^ is a typical 74-nt tRNA molecule, whereas the 85-nt tRNA^Ser^ has a longer variable loop (Supplementary Table 1). In the electrophoretic mobility shift assay (EMSA), we did not observe a clear migration shift of tRNA for rSLFN13-N or hSLFN13-N. Interestingly, the tRNA seemed degraded as the concentration of both SLFN13-N increased (Supplementary Fig. 3a). Further experiments confirmed that rSLFN13-N preferably digested tRNAs over other types of nucleic acids in a protein concentration-dependent manner, and this activity also relied on Mn^2+^/Mg^2+^ (Fig. 2a–c; Supplementary Fig. 3b, c and Supplementary Table 2). Regarding other soluble SLFN N′-domains that could be purified in our experiments, the tRNA-cleaving feature was observed for hSLFN13-N and mSLFN8-N, but not for hSLFN5-N. Moreover, hSLFN13-N exhibited the highest cleaving efficiency and specificity among these proteins (Fig. 2d). We expanded the target tRNA types, and found that hSLFN13-N was more active than rSLFN13-N in almost all cases (Supplementary Fig. 3d, Supplementary Table 1). In addition, purified full-length mSLFN1 lacks nucleolytic activity on given tRNAs (Supplementary Fig. 3e). These results exemplify the diversity of SLFNs in tRNA manipulation.

### The processing pattern of SLFN13-N for tRNAs

hSLFN13, mSLFN8 and rSLFN13 share a common cleavage product (specified as primary product, Fig. 2d), and this product appeared for all tRNA targets (Supplementary Fig. 3d), suggesting that the cleavage by SLFN13 takes place first on a specific site of tRNAs. To figure out the exact cleavage site, we generated tRNA^Ser^ molecules with differentially modified termini and tested them in the digestion assay. Extension of the 5′ end by a guanosine (5′-G-tRNA^Ser^, V1) led to larger product bands, whereas shortening of the 3′ end (tRNA^Ser^−3′-ΔGCCA, V3) had no effect (Fig. 2e). This means the original 5′ end of tRNA^Ser^ was preserved in the primary product, and the cleavage site is close to the 3′ end. When two extra bases (GC) were added to the 5′ end of the tRNA^Ser^ (5′-GC-tRNA^Ser^, V2) which extended the acceptor stem from 7 to 9 bp, the tRNA became resistant to SLFN13. Once the GCCA motif at the 3′ end was removed from this tRNA molecule (5′-GC-tRNA^Ser^−3′-ΔGCCA, V4), it became cleavable again (Fig. 2e). These results indicate that the length of the paired bases of the tRNA acceptor stem is important for the selectivity of SLFN13. By comparing the migration rate of SLFN13-digested tRNA^Ser^ with designated markers in electrophoresis assay, we found that the primary cleavage takes place on the acceptor stem of the substrate tRNA and most likely removes 11 nt at the 3′-terminus (Fig. 2f, g). For some tRNAs, a ‘secondary’ cleavage product could be observed, which seems to be derived from further digestion by SLFN13-N as it never appeared alone (Fig. 2a–e; Supplementary Fig. 3b–d). According to the size and migration feature of this product, we infer that the secondary cleavage takes place on the T-loop, which dissevers another 11 or 12 nt from the primary product (Supplementary Fig. 3f).

To further investigate the selectivity of SLFN13-N on tRNAs, we assayed several types of tRNAs from various species for SLFN13-N cleavage. Most of the tRNAs were able to be digested by hSLFN13-N, whereas rSLFN13-N seemed more active to those tRNAs with longer variation loops (Supplementary Fig. 4a–d and Supplementary Table 1). Interestingly, both hSLFN13-N and rSLFN13-N lacked the endoribonuclease activity on tRNA^Sec^ that possesses a 9-bp acceptor stem (Supplementary Fig. 4c, d). This result supports our finding that the length of acceptor stem is critical for the selectivity of SLFN13-N (Fig. 2e).

### SLFN13-N is structurally distinct from other RNases

**Fig. 3:**
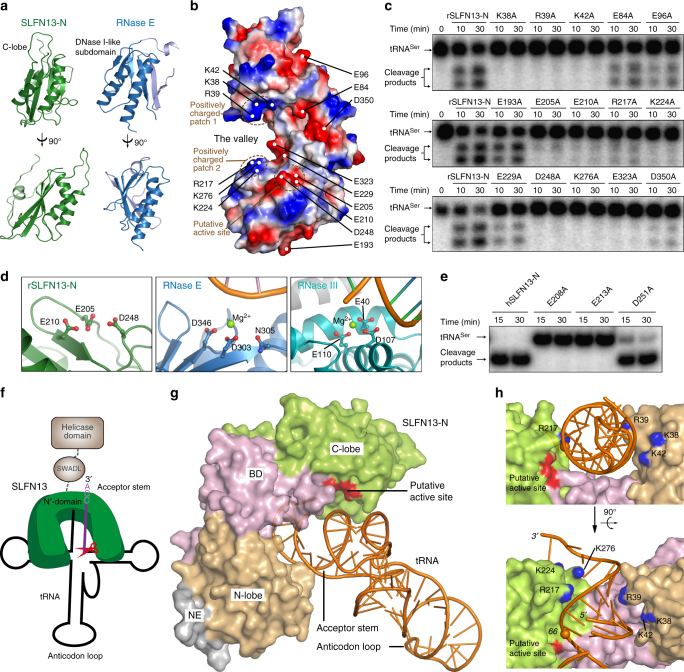
Putative catalytic site and cleavage model of SLFN13-N. **a** Structural comparison between the C-lobe of rSLFN13_14–353_ and the DNase I subdomain of RNase E (PDB ID 2BX2). For RNase E, the portion that is structurally aligned to rSLFN13_14–353_ C-lobe is shown in blue, and the rest part in light blue. **b** The electrostatic surface potentials of rSLFN13_14–353_, coloured from red (negative) to blue (positive). Two negatively charged patches and the conserved positively charged area are indicated. Positions of the residues tested in **c** are specified. **c** tRNA cleavage assay for rSLFN13_1–__353_ mutants of conserved charged residues in the presence of Mg^2+^. For each sample, 500 nM protein was used. **d** Comparison between the putative nucleolytic active site of rSLFN13-N and the actives sites of RNase E (2BX2) and RNase III (2EZ6). Note the similar tripod architecture of the three residues for each protein, which are shown as ball-and-stick models. **e** Cleavage assay of hSLFN13_1–355_ mutants at the putative active site. E208A, E213A and D251A of hSLFN13-N correspond to E205, E210 and D248 of rSLFN13-N, respectively. **f** Schematic drawing of SLFN13 cleaving a tRNA. The U-pillow-shaped SLFN13 N′-domain embraces the acceptor stem of the tRNA. The 3′ tail that is to be cleaved off the tRNA by SLFN13-N is coloured purple. **g** Structural model of SLFN13-N manipulating tRNA. rSLFN13_14–353_ is shown as surface representations and the subdomains are colour-specified. The putative active site is coloured red. The coordinate of tRNA^Gly^ (5E6M, excerpted) is used and coloured orange. SLFN13-N clamps the acceptor stem of tRNA and may have no contact with other parts of the tRNA. **h** Close view of SLFN13-N accommodating the acceptor stem. The surface positively charged residues of rSLFN13_14–353_ tested in **c** are coloured blue. Part of the tRNA is removed for clarity. The 5′ and 3′ ends of the tRNA are indicated. The phosphate atom of the 66th nucleotide of tRNA is shown as a sphere

The observed tRNA-cleaving activity of SLFN13-N prompted us to scrutinise its structure again. rSLFN13-N shows no apparent homology to known protein families, including other tRNA-cleaving enzymes in archaea (EndA), bacteria (colicin E5) or eukaryotes (PaT in yeast and angiogenin in mammals)^26–29^. In addition, rSLFN13-N also differs from Drosha^30^ and Dicer^31^ which also contain pseudo-dimeric catalytic domains (Supplementary Fig. 5a). We compared rSLFN13-N structure with existing crystal structures using the Dali server^32^. The ouput top hits included several bacterial proteins and the aligned region covered only one lobe of SLFN13 (Supplementary Fig. 5b). Apart from the uncharacterised proteins, we noticed that the lobes of rSLFN13-N are topologically related to the DNase I-like subdomain of the *E.coli* endoribonuclease RNase E^33^. The central four-stranded β-sheet and surrounded α-helices of SFLN13-N C-lobe could be substantially aligned to the DNase I-like subdomain with a root mean square deviation (rmsd) of 3.14 Å, although the latter has two extra β-strands (Fig. 3a; Supplementary Fig. 5c, d). The DNase I-like subdomain is a component of the RNase E catalytic domain that forms a homotetramer^33^. Within this tetramer, the DNase I subdomain dimerises and cleaves two single-stranded RNA at both sides of the dimer (Supplementary Fig. 5e). Compared with RNase E, native rSLFN13-N is a monomer in solution (Supplementary Fig. 2a), and has distinct overall architecture and substrate preference. Thus, although bearing putative evolutional traces from the bacterial RNases, SLFN13-N represents a novel class of endoribonuclease.

### Critical regions of SLFN13-N for tRNA cleavage

Surface electrostatic potential analysis of rSLFN13-N revealed two positively charged patches inside the valley, whose width is exactly suitable for accommodating base-paired RNA (Fig. 3b). Substitution of conserved charged residues (Lys38, Arg39, Lys42, Arg217, Lys224, Lys276) to alanine resulted in reduced nucleolytic activity, suggesting that rSLFN13-N may embrace base-paired RNA within its valley (Fig. 3c). Apart from the positively charged patches, a negatively charged area formed by conserved Glu205, Glu210 and Asp248 of the C-lobe is also observed at the rim of the valley (Fig. 3b; Supplementary Figs 6 and 7a, b). The three-carboxylate triad is reminiscent of the catalytic residues of bacterial endoribonucleases involved in RNA processing, such as RNase E and RNase III, which coordinate a hydrated Mg^2+^, possibly for the nucleophilic attack on the scissile phosphate in the RNA backbone^33,34^ (Fig. 3d). Mutating any of the three residues diminished the nucleolytic activity of rSLFN13-N and hSLFN13-N (Fig. 3c, e). Even at elevated enzyme concentration, E205A and E210A mutants were absolutely inactive, which differed from other single-point mutants (D34A, E47A, E84A, E96A, E146A, D193A, E221A, E229A, E249A, D259A, E323A, D350A) regarding exposed and relatively conserved aspartates or glutamates (Fig. 3c, e; Supplementary Fig. 7c–e). This result demonstrates, that Glu205 and Glu210 are pivotal residues for the endonucleolytic activity of SLFN13-N. In addition, this plausible active site appears only in the C-lobe but not in the N-lobe (Supplementary Fig. 6), suggesting that SLFN13 cleaves only on one site of base-paired RNA. This is in accordance with the results of tRNA cleavage assays (Fig. 2e).

### Mechanistic model of tRNA cleavage by SLFN13-N

Based on the biochemical analysis, we propose that SLFN13-N clamps the tRNA acceptor stem and cleaves in a site-specific and sequence-independent manner, which is likely controlled by the interplay between the inner shape of the valley and the tertiary structure of the tRNAs (Fig. 3f). To illustrate this special mechanism, we generated a structural model of SLFN13-N–tRNA complex (Fig. 3g). In this model, the acceptor stem of the tRNA is perfectly docked in the valley of SLFN13-N, where the protruding positively charged residues support the tRNA backbone, and putative active site of SLFN13-N is right next to the predicted cleavage site between the 65th and 66th nucleotide of tRNA. The 5′ end of tRNA is tightly packed in the valley, leaving insufficient space for an extra base (Fig. 3h). This may explain why SLFN13-N does not cleave the V2-tRNA^Ser^ that has a 9-bp acceptor arm (Fig. 2e). This reaction would cause the loss of the CCA tail, hence the amino-acylation of tRNA molecules.

### SLFN13 restricts cellular tRNA and rRNA

**Fig. 4:**
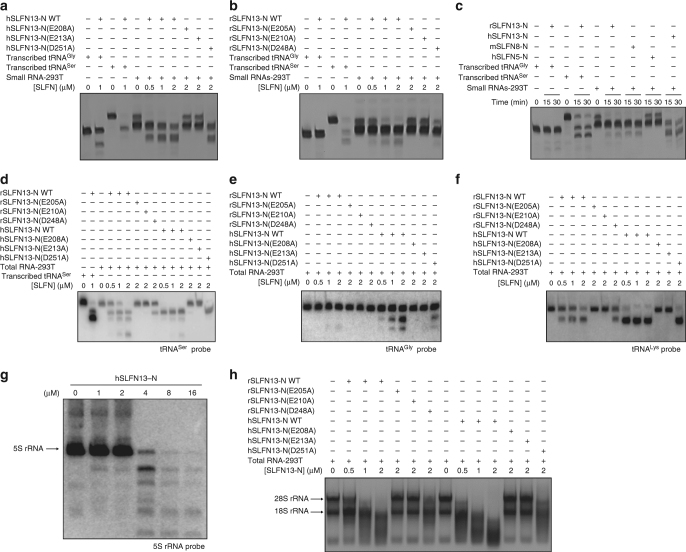
SLFN13-N cleaves native tRNA and rRNA. **a**, **b** Cleavage assay of hSLFN13-N (**a**) and rSLFN13-N (**b**) on native tRNAs in the presence of Mg^2+^. Small RNA-293T denotes total small RNA extracted from 293T cells that is mainly constituted of tRNAs. tRNA^Ser^ and tRNA^Gly^ were used as controls to indicate molecular weight. **c** Cleavage assay of different SLFN-Ns on native tRNAs. **d**–**f** Cleavage assay on specific native tRNAs. The substrates and products were identified by Northern blot using [γ-^32^P]-labelled probes targeting tRNA^Ser^ (**d**), tRNA^Gly^ (**e**) or tRNA^Lys^ (**f**). **g** Cleavage assay for hSLFN13-N on 5S rRNA. 5S rRNA and products were identified by Northern blot. **h** Cleavage assay on native rRNAs. 4 μg total RNA extracted from 293T cells was used for each reaction, which mainly contains rRNAs

To understand whether SLFN13-N is able to digest natural tRNAs carrying various post-transcriptional modifications, total tRNAs isolated from HEK-293T (293T) cells or HeLa cells were applied to cleavage assays. When incubated with hSLFN13-N, mSLFN8-N or rSLFN13-N, the total tRNA showed a degradation pattern similar to the in vitro experiments in a protein concentration-dependent manner (Figs 2a, 4a–c; Supplementary Fig. 8a–c). Northern blot assays confirmed that several types of tRNA were susceptible to hSLFN13-N and rSLFN13-N digestion (Fig. 4d–f; Supplementary Table 3). In addition, SLFN13-N was able to digest the 5S, 18S and 28S rRNA extracted from 293T cells or HeLa cells (Fig. 4g, h; Supplementary Fig. 8d). We surmise, that SLFN13-N cuts rRNAs at base-paired regions that are topologically similar to the acceptor stem of tRNAs.

**Fig. 5:**
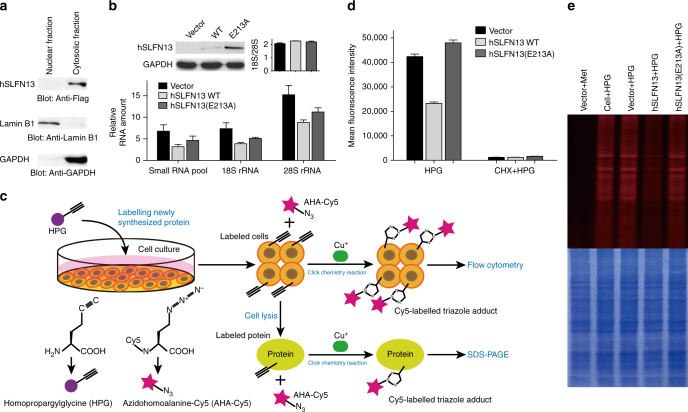
hSLFN13 disrupts translational machinery. **a** Subcellular localisation of hSLFN13. **b** hSLFN13 restricts tRNAs and rRNAs in cells. The small RNA pool is mainly constituted of tRNAs and 5S/5.8S rRNA (See Supplementary Fig. 8f). Expression of hSLFN13 WT and hSLFN13(E213A) was confirmed by Western blot. Note the prominent restriction of cellular RNAs caused by relatively low level of overexpressed hSLFN13. Error bar indicates s.d. (*n* = 3). **c** The experimental process of the FUNCAT-click chemistry assay. **d** FUNCAT assay and subsequent flow cytometry examination for Cy5 fluorescence showing that hSLFN13 inhibits protein synthesis in living cells. **e** hSLFN13 inhibits protein synthesis. Upper: fluorescent image of the SDS-PAGE gel showing the newly synthesised proteins labelled by FUNCAT assay. Lower: same gel stained with Coomassie Blue showing the loading consistency

Next, we investigated hSLFN13 in vivo. Although several other Schlafen family members of subgroup III were reported to carry a nuclear localisation signal (NLS)^22^, hSLFN13 does not seem to have any NLS (Supplementary Figs 1a and 6). Overexpressed hSLFN13 showed a preference for cytoplasmic distribution in 293T cells and HeLa cells, therefore, it should be accessible to mature tRNAs and ribosomes (Fig. 5a; Supplementary Fig. 8e). Indeed, the tRNA and rRNA levels in hSLFN13-expressing cells were markedly reduced, which is apparently associated with its nucleolytic activity (Fig. 5b; Supplementary Fig. 8f). We then investigated the effect of hSLFN13 on protein synthesis based on fluorescent non-canonical amino acid labelling (FUNCAT). Newly synthesised proteins were labelled using homopropargylglycine (HPG) and detected via click chemistry (Fig. 5c). In hSLFN13-transfected 293T cells, the amount of newly synthesised proteins was greatly diminished (Fig. 5d, e). These results prove that hSFLN13 can disrupt translational machinery and retard protein synthesis by restricting cytoplasmic tRNA and rRNA.

### SLFN13 restricts HIV in a nucleolytic activity-dependent manner

**Fig. 6:**
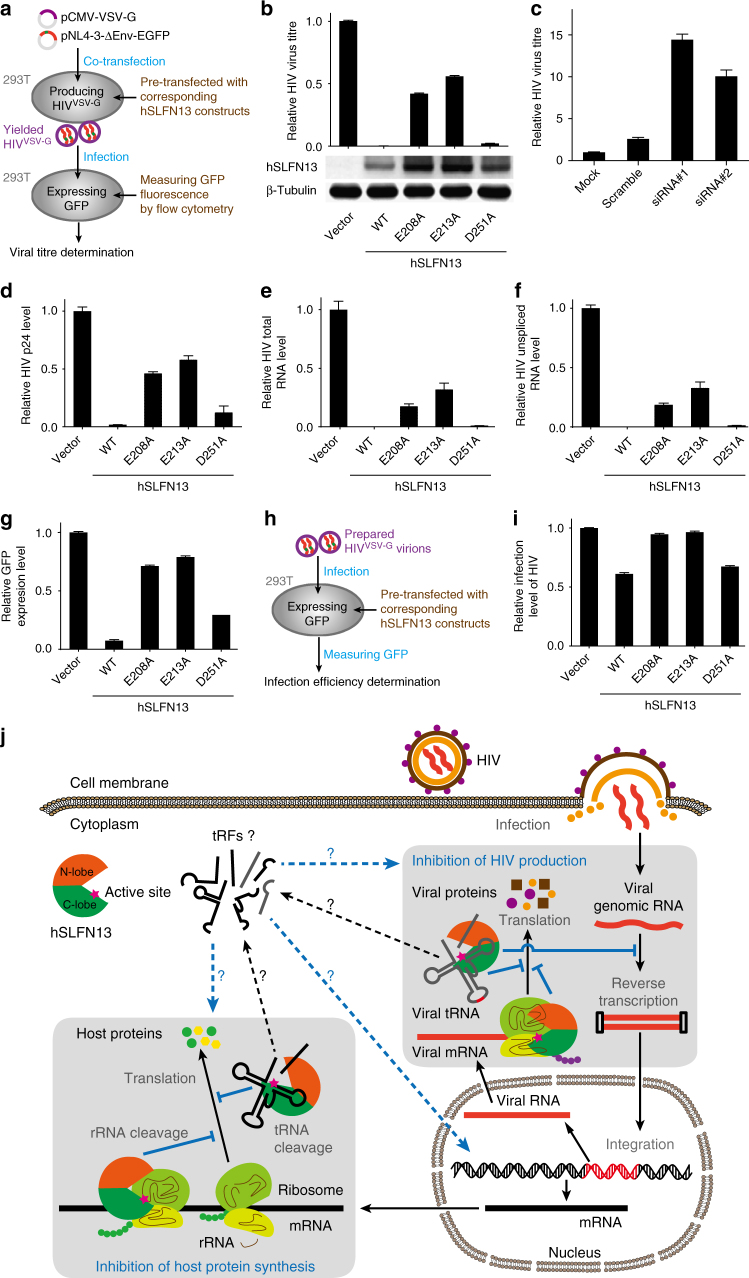
hSLFN13 restricts HIV production. **a** The experimental process of titre assay for VSV-G pseudotyped HIV (HIV^VSV-G^). **b** Viral production was assayed by titrated infection and the GFP level. Expression of hSLFN13 WT and mutants was confirmed by Western blot. Error bar indicates s.d. (*n* = 3). **c** Knock-down of endogenous SLFN13 increased viral titre of 293 cells. **d** Viral particle content in supernatants was analysed by p24 ELISA. **e**, **f** Extracellular vRNA concentration was determined by qPCR for total vRNA (**e**) and unspliced vRNA (**f**). **g** hSLFN13 suppressed GFP production from the viral plasmids. **h** The experimental process of the viral infection assay. **i** Examination for the infection efficiency of HIV^VSV-G^ in cells pre-transfected with hSLFN13 WT or mutants. **j** Proposed protein synthesis inhibition and anti-HIV mechanism of hSLFN13

Several subgroup III SLFNs are suggested to restrict different viruses^21,23^. While hSLFN13 was reported to moderately inhibit influenza virus, it is intriguing as to whether SLFN13 also inhibits other types of viruses. Expression of SLFN13 was not inducible by IFNɑ/β/γ treatment in 293T cells, but could be stimulated by the infection of VSV-G pseudotyped HIV-1 (HIV^VSV-G^) (Supplementary Fig. 9a, b). To confirm whether SLFN13 is capable to restrict HIV, we transfected 293T cells with pNL4-3-ΔEnv-EGFP (encoding essential HIV-1 proteins but the coding sequence for HIV-1 Envelope was partially replaced by the cDNA of EGFP) and pCMV-VSV-G (encoding the vesicular stomatitis virus G glycoprotein, or VSV-G that serves as a surrogate viral envelope protein), and measured the titre of VSV-G pseudotyped HIV-1 (HIV^VSV-G^) produced from these cells in the presence of hSLFN13/hSLFN13-N constructs (Fig. 6a). After being transfected with wild-type (WT) hSLFN13, these cells failed to yield HIV^VSV-G^ titre, indicating the restriction of HIV (Fig. 6b). In agreement with this, knock-down of *SLFN13* dramatically weakened HIV resistance of 293 cells (Fig. 6c; Supplementary Fig. 9c,d). Apart from HIV, we also tested antiviral activity of SLFN13 on viruses belonging to *Herpesviridae* and *Flaviviridae*. hSLFN13 failed to confer efficient resistance against herpes simplex virus (HSV), and only moderately restricted Zika virus (Supplementary Fig. 9e, f).

The nucleolytic-deficient mutations E208A and E213A dramatically decreased the anti-HIV activity of hSLFN13, while the less destructive mutant D251A remained highly active (Fig. 6b). This anti-HIV tendency of hSLFN13 mutants is congruent with their behaviours in the tRNA cleavage assays (Figs 3e, 4a–c; Supplementary Fig. 7d), manifesting the crucial role of hSLFN13's nucleolytic activity in HIV restriction. Moreover, hSLFN13-N also efficiently reduced viral titre, whereas hSLFN13-N(E208A) and hSLFN13-N(E213A) showed much weaker anti-HIV activity compared to their full-length counterparts (Supplementary Fig. 9g), suggesting that the SWADL domain and/or helicase domain may also contribute to the anti-HIV activity of hSLFN13.

### SLFN13 blocks synthesis of viral proteins

To discern whether hSLFN13 reduced the number or the viability of the released virus, we measured viral protein and viral RNA (vRNA) levels in the supernatant of hSLFN13/hSLFN13-N-transfected, HIV^VSV-G^-producing cells. Extracellular concentration pattern of p24 capsid, total vRNA or unspliced vRNA was consistent with the titre results, suggesting that hSLFN13 diminished the number of released viral particles (Fig. 6d–f; Supplementary Fig. 9h–j and Supplementary Table 4). Moreover, hSLFN13/hSLFN13-N suppressed the fluorescence from 293T cells transfected with viral vector pNL4-3-ΔEnv-EGFP (Fig. 6g; Supplementary Fig. 9k), which was strong evidence for the disrupted synthesis of viral proteins. To clarify whether this observation resulted solely from overall protein synthesis arrest caused by hSLFN13 or hSLFN13-N, we compared the fluorescence of various EGFP-containing plasmids co-transfected with hSLFN13/hSLFN13-N in 293T cells. Plasmids with HIV promoter were obviously more susceptible than the control plasmid (Supplementary Fig. 9l, m), illustrating the preference of hSLFN13 over HIV-related protein synthesis. This preference is possibly resulted from the cleavage by SLFN13 on the HIV-derived sequences of the transcripts, as they contain stem loops which structurally resemble the SLFN13-targeting regions of tRNAs (Fig. 2g). In addition, we also checked whether hSLFN13 hinders HIV^VSV-G^ infection (Fig. 6i). Although 293T cells pre-transfected with hSLFN13/hSLFN13-N showed a decreased GFP level (Fig. 6i; Supplementary Fig. 9n), this result may be attributable to the restriction of global protein synthesis. Thus, it is currently tentative to conclude SLFN13 affects the infection of HIV.

## Discussion

Our study has revealed SLFN13 as a novel type of eukaryotic tRNA/rRNA endoribonuclease with several features. First, its unique pseudo-dimeric U-pillow-shaped architecture allows for the docking of base-paired RNA. Second, SLFN13 seems to lack the discrimination over tRNA types but relies on the recognition of the secondary or tertiary structure. Third, according to the effects of overexpressed SLFN13 in translational control of cell lines, it is possible that upon certain cellular stresses, induced SLFN13 inhibits protein synthesis by directly digesting cytoplasmic tRNA and rRNA, so as to realise a fast stress response. Fourth, SLFN13 is so far the only eukaryotic endoribonuclease that has been proven to preferably cleave at the acceptor stem of mature tRNAs. Many other cytosolic tRNA processors target the anti-codon loop, such as stress-induced mammalian angiogenin^15,35^ and yeast Rny1p^14^, as well as *E. coli* PrrC and colicin D/E5 involved in the bacterial defence system^9,36–38^. In bacteria, the secreted bacterial toxin CdiA family involved in contact-dependent growth inhibition was also reported to cut the acceptor stem of certain tRNAs^39^. However, unlike SLFN13, the in vitro nucleolytic activity of CdiA family proteins is dependent on the association with elongation factor Tu (EF-Tu) and the presence of GTP^40^. According to these properties, we propose a novel nomenclature for SLFN13: RNase S13.

The nucleolytic activity of hSLFN13 is crucial for its anti-HIV potency. The cleavage of cytoplasmic tRNA and rRNA by hSLFN13 may cause global translation inhibition that suppresses both viral replication and cell viability to confine viruses (Fig. 6j). A similar mechanism was discovered for the suicidal tRNase PrrC in *E. coli*, which cleaves its own tRNA^Lys^ in response to bacteriophage T4 infection^36^. This may explain a recent observation that marmoset SLFN11 blocks synthesis of both host and viral proteins^41^. In addition, tRNA degradation may also negatively impact the reverse transcription of HIV which requires host tRNA^Lys^ as the primer^42^.

We observed the preference of SLFN13 for blocking synthesis of viral proteins as compared to endogenous ones. As the key determinant of tRNA cleavage by SLFN13 is the secondary structure but not the anti-codon sequence, we assume that the preference for HIV protein synthesis of SLFN13 may not stem from the codon-usage-based mechanism reported for SLFN11^23^. On the other hand, HIV genome contains rich stem loop regions that are essential for the replication and packaging of the virus^43,44^. SLFN13 may directly digest some of these stem loops to specifically disrupt the life cycle of HIV. The tRNA-derived fragments (tRFs) have been suggested to constitute a new group of functional none-coding RNAs^13^. These tRFs, generated by certain tRNases including angiogenin, were found to be involved in cellular stress response and cancer progression by means of regulating transcription and translation in cells^45–48^. This mechanism might also apply to the tRNA fragments generated by SLFN13 which retards protein synthesis. Overall, more targets of SLFN13 need to be identified in order to understand its detailed antiviral mechanism.

We found that hSLFN5-N and mSLFN1, albeit both retaining the key catalytic residues (corresponding to Glu205 and Glu210 of rSLFN13), did not cleave the given tRNAs (Fig. 2d). Compared to rSLFN13, hSLFN5 lacks two positively charged residues that were proven critical for tRNA/rRNA digestion (i.e. Lys42 and Arg217 of rSLFN13), whereas the equivalents of rSLFN13 Arg39, Lys42 and Lys276 are absent in mSLFN1 (Supplementary Fig. 6). In contrast, hSLFN13, mSLFN8, as well as rabbit SLFN14 which was reported active for tRNA/rRNA cleavage^24^, showed higher sequence consistency in the positively charged patches (Fig. 3b; Supplementary Fig. 6). We conclude that the distribution of positively charged residues inside the valley of the N′-domain may define the capability and selectivity in tRNA/rRNA cleavage, as well as the antiviral spectrum, for different SLFNs. Although more enzymatic studies and characterisation of the SWADL domain and helicase domain are needed for different SLFNs, it is conceivable that tRNA/rRNA manipulation may be a hallmark of the Schlafen family, and the difference in their N′-domains may be one of the factors that define the functional diversity of these proteins.

Regarding the functional diversity of the Schlafen family, other than the restriction of viruses, the inhibition of cell proliferation has been suggested as a prominent character for many members. For example, hSLFN5 was found to negatively regulate the anchorage-independent growth and invasion of malignant melanoma cells^19^. Mouse SLFN1, SLFN2, SLFN3 and SLFN5 were all shown as proliferation suppressors when individually expressed in NIH3T3 murine fibroblasts, T cells or various human tumour cells^18,20,25,49–51^. In addition, mouse SLFN2 was also reported to play an important role in maintaining the quiescence of T cells and monocytes during immune response^52^. Given the prevalence of the N′-domain in SLFNs and the apparent relationship between the translational level and cell proliferation/growth, it is tempting to speculate that SLFNs exert the aforementioned functions through translational regulation. Thus, our study provides valuable clues for investigating the functional mechanisms of the versatile Schlafen family in the future.

## Methods

### Molecular cloning

cDNAs of SLFN-N constructs, including those for crystallisation (rSLFN13_14–353_) and cleavage assays (rSLFN13_1–353_, mSLFN8_1–359_, hSLFN13_1–355_ and hSLFN5_12–334_), were individually cloned into a modified pET28 vector. Details of major constructs are illustrated in Supplementary Fig. 1b. For eukaryotic expression, full-length hSLFN13 (hSLFN13_FL_) and hSLFN13_1–355_ were individually cloned into the pCAGGS vector with an N-terminal Flag-tag. Indicated mutants were prepared based on corresponding recombinant plasmids by site-directed mutagenesis. In the fluorescence assay, pEGFP-C1 vector was used to generate recombinant plasmid expressing GFP fused hSLFN13_FL_.

### Protein expression and purification

Recombinant proteins containing an N-terminal His_6_-tag followed by a cleavage site for PreScission protease (PSP) were expressed in *Escherichia coli* (*E. coli*) *Rosetta* (DE3). Transformed bacteria were cultured at 37 °C in Terrific Broth (TB) medium before induced with 0.1 mM isopropyl-1-thio-β-d-galactopyranoside at an OD_600_ nm of 0.6, and grown overnight at 16 °C. Cells were lysed in 50 mM Tris-HCl, pH 8.2, 300 mM NaCl, 30 mM imidazole, 1 μM DNase I, 1 mM phenylmethanesulfonylfluoride (PMSF) and 2 mM β-mercaptoethanol (β-ME) using a cell disruptor (JNBIO) and subjected to centrifugation at 40,000 × *g* for 50 min. The supernatant was filtered and applied to the first Ni-NTA column (GE Healthcare) equilibrated with binding buffer 1 containing 20 mM Tris-HCl, pH 8.2, 300 mM NaCl, 30 mM imidazole and 2 mM β-ME. After washed with wash buffer containing 20 mM Tris-HCl, pH 8.2, 300 mM NaCl, 70 mM imidazole and 2 mM β-ME, proteins were eluted with elution buffer containing 20 mM Tris-HCl, pH 8.2, 300 mM NaCl, 300 mM imidazole and 2 mM β-ME. Except for rSLFN13_14–353_, the eluted protein was incubated with glutathione S-transferase (GST)-fused PSP to remove the His_6_-tag and dialysed overnight against binding buffer 2 containing 20 mM Tris-HCl, pH 8.2, 300 mM NaCl and 2 mM β-ME. After dialysis, PSP was removed using a GST column. The protein was re-applied to the second Ni-NTA column equilibrated with binding buffer 2. Binding buffer 1 was used to elute the proteins which were subsequently loaded onto a Superdex200 16/60 column (GE Healthcare) equilibrated with gel filtration buffer containing 20 mM Tris-HCl, pH 8.2, 150 mM NaCl and 1 mM dithiothreitol (DTT). The proteins were eluted in a discrete peak corresponding to a molecular mass of approximately 40 kDa. For rSLFN13_14–353_, the protein was directly applied to gel filtration using a Superdex200 16/60 column (GE Healthcare) equilibrated with buffer containing 20 mM HEPES, pH 7.0, 150 mM NaCl and 1 mM DTT after elution from the first Ni-NTA. The proteins were eluted in a discrete peak corresponding to a molecular mass of approximately 80 kDa. Cell lysis and protein purification were both performed at 4 °C. The yields of SLFN13-N mutants used for endoribonuclease study were comparable with that of wide-type protein. The selenomethionine (SeMet) derivative of rSLFN13_14–353_ was insoluble. We therefore made an alanine screening mutagenesis for each methionine of rSLFN13_14–353_ to look for soluble constructs, which resulted in rSLFN13_14–353_(M179A) as an optimal candidate. SeMet-substituted rSLFN13_14–353_(M179A) was expressed as previously described^53^ and purified as native rSLFN13_14–353_, except that 5 mM instead of 2 mM β-ME was used in first Ni-NTA purification, and 2 mM instead of 1 mM DTT was used in gel filtration.

### Protein crystallisation

Crystallisation trials by the sitting-drop vapour-diffusion method were performed at 4 °C. rSLFN13_14–353_ (18 mg ml^−1^ in buffer containing 20 mM HEPES, pH 7.0, 150 mM NaCl and 1 mM DTT) was mixed with an equal volume of reservoir solution containing 20% PEG6000, 100 mM MES, pH 6.5 and 850–950 mM lithium chloride. Crystals of the native protein appeared after 5 days and grew to a maximum size in 10 days. Crystals of SeMet-substituted rSLFN13_14–353_(M179A) were obtained in the same conditions as native rSLFN13_14–353_. For data collection, crystals of native and Se-Met protein were flash-frozen in the reservoir solution containing extra 20% PEG200.

### Structure determination

X-ray diffraction data set of native rSLFN13_14–353_ was collected from a single crystal at beamline BL19U1 of the Shanghai Synchrotron Radiation Facility (SSRF), and one data set of SeMet-substituted rSLFN13_14–353_(M176A) at the selenium peak wavelength was collected at beamline BL17U1 of SSRF^54^. Data sets were processed with the XDS suite^55^ and HKL-2000 programme suite^56^. Initial phases were obtained by the single-anomalous dispersion method and refined using Phaser^57^. In the calculated electron density, the main chains and many side chains were clearly traceable. Owing to the high quality of the density map, an initial model was manually built with COOT^58^, where the positions of 31 selenium atoms in the asymmetric unit were used to assign the sequences of four peptide chains. The model was further refined with Refmac^59^ and Phenix^60^. Structural validation was carried out using MolProbity^61^. Structural illustrations were prepared using the PyMOL Molecular Graphic Systems (version 0.99, Schrödinger LLC; http://www.pymol.org/). X-ray data collection and refinement statistics can be found in Extended Data Table 1. The Ramachandran statistics determined by PROCHECK^62^ are as follows: 94.48% in favoured region, 4.97% allowed, 0.55% outlier. To confirm the incorporation of zinc, X-ray fluorescence scan over the rSLFN13_14–353_ crystal was carried out at SSRF, and the excitation energy was 9679 eV.

### tRNA substrate preparation

Preparation of tDNA transcription template and in vitro transcription of tRNAs was performed as previously described^63^. To prepare [α-^32^P]-labelled tRNA, 250 ng µl^−1^ tDNA transcription template was mixed with 2 mM of each NTP, 3.4 μM [α-^32^P] ATP (6000 Ci/mmol, PerkinElmer), 20 mM Tris-HCl, pH 8.0, 150 mM NaCl, 20 mM MgCl_2_, 5 mM DTT, 1 mM spermidine and 0.3 µM T7 RNA polymerase at 37 °C for 1 h. The labelled tRNA product was subsequently purified with a Zymo-Spin IC Column (Zymo Research) and eluted with 200 μl TE buffer containing 20 mM Tris-HCl, pH 8.0 and 1 mM EDTA. The eluted tRNA was aliquoted and stored at −20 °C if not immediately used. Sequences of the tRNA transcripts used in this study were listed in Supplementary Table 1.

### EMSA

rSLFN13_1–353_ or hSLFN13_1–355_ of increasing concentrations was individually incubated with 5 μM tRNA^Gly(CCC)^ or tRNA^Ser(UAG)^ in buffer containing 40 mM Tris-HCl, pH 8.5, 20 mM KCl, 2 mM MgCl_2_, 2 mM DTT and 5% glycerol for 30 min at 25 °C. After the incubation, each sample was mixed with loading buffer containing 3% glycerol and 0.05% bromophenol blue before loaded onto a 6% native polyacrylamide gel containing 40 mM Tris-acetate, pH 8.5. The gel was pre-run at 4 °C for 45 min at 14.0 V cm^−1^ with running buffer containing 40 mM Tris-acetate, pH 8.5. After electrophoresis the gel was stained with ethidium bromide (EB) and visualised by UV light.

### Cell culture

Unless otherwise specified, HeLa, HEK-293, HEK-293T, Vero and A549 cells were individually maintained at 37 °C in high glucose Dulbecco's modified Eagle medium (DMEM) supplemented with 10% fatal calf serum, 100 U ml^−1^ penicillin and 100 μg ml^−1^ streptomycin. Albopictus C6/36 cells, used for propagation of the Zika virus, were grown in DMEM supplemented with 10% fetal calf serum at 28 °C. All cell lines are free of mycoplasma. HeLa, 293, 293T and A549 cell lines have been authenticated by genotyping.

### In vitro cleavage assay

For substrate screening assay, 2 µM rSLFN13_1–353_ was individually incubated with 1 µM each ssDNA, ssRNA, dsDNA, dsRNA, DNA/RNA duplex, stem-loop RNA or tRNA^Ser^ in the cleavage buffer containing 40 mM Tris-HCl, pH 8.0, 20 mM KCl, 4 mM MgCl_2_ and 2 mM DTT for 30 min at 37 °C. The sequences of the nucleic acids used in this experiment are summarised in Supplementary Table 2.

For cleavage assays on in vitro transcribed tRNAs, either [α-^32^P]-labelled or non-labelled tRNA transcripts were used as substrates. For radiolabelled tRNAs to be detected by autoradiography, protein of increasing concentration was mixed with labelled tRNA (>1 × 10^4^ cpm) in the cleavage buffer at 37 °C. Aliquots were taken at different time points as indicated, and the reaction was terminated by adding 2× formamide gel-loading buffer (95% w/v formamide, 50 mM EDTA). The cleavage products were analysed by electrophoresis at 250 V by a 12% denaturing urea-polyacrylamide gel (Urea-PAGE) containing 7.5 M urea with 1× TBE (89 mM Tris, 89 mM borate and 2 mM EDTA) as the running buffer. All the gels were exposed to a phosphorimager plate overnight before scanned with a Typhoon scanner (GE Healthcare). For non-labelled tRNA substrates, 1 µM protein was mixed with 2 µM tRNA^Ser^ in the cleavage buffer at 37 °C. Subsequent experimental procedures were the same as those for labelled tRNA substrates. After electrophoresis, the gel was stained with EB and visualised by UV light.

For cleavage assays on native RNAs, total small RNA was extracted from cultured 293T or HeLa cells using mirVana miRNA Isolation kit (Ambion) following the small RNA isolation protocol. An aliquot of 1 µM or indicated amount of protein was incubated with 0.5 μM control tRNAs or 50 ng µl^−1^ total small RNA in the cleavage buffer for 30 min or indicated time at 37 °C. The cleavage products were analysed by electrophoresis. Total RNA was extracted from cultured 293T or HeLa cells using TRI Reagent (Sigma-Aldrich). Protein of indicated concentrations was incubated with 20 ng µl^−1^ tRNA^Ser^ control, or 0.4 µg µl^−1^ (for EB staining) or 1 µg µl^−1^ (for Northern blot) total RNA in the cleavage buffer for 30 min at 37 °C. Cleavage products were analysed by electrophoresis, and then either stained with EB or subjected to Northern blot.

### Northern blot analysis

The cleavage reaction to be analysed by Northern blot was denatured by heating at 80 °C for 10 min in a 2× formamide gel-loading buffer, followed by quick chill on ice. These samples were separated by Urea-PAGE and subsequently electroblotted onto the Hybond N+ membranes (GE Healthcare) at 250 mA for 3 h in 1× TBE buffer. After air-dried for 10 min, the RNA samples were fixed to the membrane by UV cross-linking. The membrane was pre-hybridised in the hybridisation buffer containing 0.2% Ficoll (Mr = 40,000), 0.2% polyvinyl-pyrrolidone (Mr = 40,000), 0.2% bovine serum albumin (BSA), 50% formamide, 50 mM Tris-HCl, pH 7.5, 1 M NaCl, 0.1% sodium pyrophosphate and 0.1 mg ml^−1^ denatured salmon sperm DNA at 42 °C for 3 h. The hybridisation was carried out at 42 °C overnight in the presence of [γ-^32^P]-ATP (6000 Ci/mmol, PerkinElmer) DNA oligonucleotide probes which were previously end-labelled using T4 polynucleotide kinase. The probe sequences were complementary to the 5′ end of specified tRNAs and 5S rRNA (see Supplementary Table 3 for details). The hybridised membrane was washed three times with 1× SSC buffer (150 mM NaCl, 15 mM sodium citrate, pH 7.0 and 0.1% SDS) at 42 °C for 30 min each. The blots were exposed to a phosphorimager plate overnight before scanned with a Typhoon scanner (GE Healthcare).

### Western blot analysis

Following antibodies were used in for Western blot assays in this paper: anti-Flag antibody (Sigma-Aldrich, F1804, 1:1000 used), anti-β-tubulin antibody (ProteinTech, 66240-1-Ig, 1:2000 used), anti-Lamin B1 (ProteinTech, 66095-1-Ig, 1:1000 used), anti-GAPDH (60004-1-Ig, 1:5000 used), HRP-conjugated anti-mouse secondary antibody (Cell Signaling Technology, 7076, 1:3000 used) and HRP-conjugated anti-rabbit secondary antibody (ProteinTech, SA00001-2, 1:3000 used). The cell pellets after transfection were lysed in ice-cold lysis buffer (20 mM HEPES, pH 7.9, 1 mM EDTA, 400 mM NaCl, 10 mM KCl, 1% Nonidet P-40 and 20% Glycerol) containing protease inhibitor cocktail and PMSF. After centrifugation at 12,000 × *g* for 15 min, the supernatant separated by 10% SDS-PAGE, transferred to PVDF membranes (Thermo Scientific). After an 1 h incubation with blocking buffer (5% BSA), the membrane was incubated with corresponding antibodies overnight at 4 °C. The membrane was washed and incubated with HRP-conjugated secondary antibodies. The membranes were washed again, developed with ECL Western Blotting Detection Reagents (Thermo Scientific) and visualised with ChemiDoc^TM^ Touch Imaging System (Bio-Rad).

### Subcellular localisation analysis

For fluorescence assay, 293T cells and HeLa cells grown on round glass slides in 24-well plates were transfected with pEGFP-C1-hSLFN13_FL_ using Lipofectamine 2000 (Thermo Scientific). After 24 h, the cells were fixed with 4% paraformaldehyde (PFA) in phosphate buffered saline (PBS, pH 7.4) at room temperature for 10 min and washed with PBS for three times. Fixed cells were stained with 4′,6-diamidino-2-phenylindole (DAPI, Sigma-Aldrich) for nuclear staining and then mounted in antifade mounting medium (Thermo Scientific). Slides were viewed with an FV1000 fluorescent microscope (Olympus).

For analysis by Western blot, 293T cells were transfected with pCAGGS-hSLFN13_FL_. After 24 h, cells were collected, and NE-PER Nuclear and Cytoplasmic Extraction Reagents (Thermo Scientific) was used to separate nuclear and cytosolic fractions after cell lysis according to manufacturer’s instructions. The two fractions were then individually subjected to Western blot analysis.

### Quantification of cellular RNA

A total of 1 × 10^5^ 293T cells transfected with empty pCAGGS vector, pCAGGS-hSLFN13_FL_ or pCAGGS-hSLFN13_FL_(E213A) were individually sorted by flow cytometry. Artificial spike-in RNA (1000 nt) was added to the sorted cells as control before total RNA was extracted using TRI Reagent (Sigma-Aldrich). Total RNA was pooled by size and quantified using Bioanalyzer and RNA 6000 Pico Kit (Agilent) following the manufacturer’s protocol.

### FUNCAT assay and click chemistry

293T cells transfected with empty pCAGGS vector, pCAGGS-hSLFN13_FL_ or pCAGGS-hSLFN13_FL_(E213A) were re-transfected with corresponding plasmids 12 h after initial transfection. 36 h after initial transfection, cells of 80% confluence were washed twice in pre-warmed PBS and then starved in serum-free DMEM lacking methionine (SFM) for 1 h (Thermo Scientific). Following the starvation, 4 mM l-HPG (Click Chemistry Tools) or 4 mM methionine was appended into SFM for a 4-h pulse as described earlier^64^.

For flow cytometry analysis, the collected cells were fixed in 0.5 ml of 1% PFA in PBS for 15 min on ice and permeabilised in PBS containing 0.1% saponin (Sigma-Aldrich) for 5 min at room temperature as described^65^. After labelled by copper-catalysed azide-alkyne cycloaddition (CuAAC), cells were washed and resuspended in PBS, and immediately analysed by flow cytometry. As an additional negative control, 100 μg ml^−1^ translational elongation inhibitor cycloheximide (CHX, Sigma-Aldrich) was added 30 min before HPG incorporation. Cy5 fluorescence signal was detected using a CytoFLEX flow cytometer (Beckman Coulter).

For SDS-PAGE analysis, cells were collected and lysed in ice-cold lysis buffer (20 mM HEPES, pH 7.9, 1 mM EDTA, 400 mM NaCl, 10 mM KCl, 1% Nonidet P-40 and 20% Glycerol) containing protease inhibitor cocktail and PMSF. Protein concentration was determined using Pierce BCA Protein Assay Kit (Thermo Scientific). HPG incorporated protein lysates were labelled selectively with Cy5-conjugated azide by CuAAC^66,67^. The click reaction was performed following the protocol from Click Chemistry Protein Reaction Buffer Kit (Click Chemistry Tools): 70 μl soluble protein lysates (1.5–4 μg μl^−1^) incorporated with HPG or methionine were sequentially mixed with 90 µl Reaction Buffer, 1 μl Cy5-azide (8 mM), 10 µl Tris(3-hydroxypropyltriazolylmethyl)amine (THPTA, 100 mM), 10 µl CuSO_4_ (20 mM) and 10 µl sodium ascorbate (300 mM). The mixture was rotated end-over-end in dark for 30 min at room temperature to generate a stable triazole adduct, and then subjected to 1× Laemmli buffer. After heated at 70 °C for 10 min, the samples were resolved by SDS-PAGE and imaged using a pharos FX^TM^ Plus Molecular Imager (Bio-Rad). Loading consistency was confirmed by staining the gels with Coomassie Blue after fluorescent imaging.

### Assay for HIV, HSV and Zika virus production

All HIV-related assays in this study were based on HIV-1. To analyse HIV retrovirus production, pNL4-3-ΔEnv-EGFP vector and pCMV-VSV-G packaging vector were transfected into the 293T cells which were pre-transfected with empty pCAGGS vector, pCAGGS-hSLFN13_FL_, pCAGGS-hSLFN13_1–355_ or corresponding mutants using Lipofectamine 2000 (Thermo Scientific) according to the manufacturer’s instructions. The old culture medium was replaced with fresh culture medium at 6 h post-transfection. Subsequently, the supernatants were collected at 48 h post-transfection and cleared by centrifugation. The amount of (infectious) virus particles in the supernatants was determined via infection assay, quantitative (q)PCR of viral RNA (total RNA and unspliced RNA) and HIV p24 ELISA (Supplementary Table 4). For infection assays, pre-plated 293T cells were infected with serially diluted supernatants for 6 h. At 24 h post-infection, the 293T cells were examined for GFP expression by flow cytometry. HIV viral RNA was extracted from the virus supernatants and quantified. The amount of p24 was measured using an HIV-1 p24 ELISA Kit (Clonetech) according to the manufacturer’s instructions. The expression of intracellular GFP was measured by flow cytometry.

For HIV package assays in 293 cells, pNL4-3-ΔEnv-EGFP vector and pCMV-VSV-G packaging vector were transfected into the 293 cells which were pre-transfected with scramble-siRNA, SLFN13-siRNA#1 or SLFN13-siRNA#2. The supernatants were collected at 48 h post-transfection and measured by infecting pre-plated 293T cells. The mRNA level of hSLFN13 was measured by qPCR (primers are listed in Supplementary Table 4).

The GFP fused HSV is a gift from Prof. G. F. Gao. For virus preparation, pre-plated Vero cells were infected with HSV (MOI = 0.03) in serum-free DMEM for 2 h. 48–72 h after the infection, the Vero cells became suspension and were collected. The virus-containing lysates were generated by freeze/thaw cycles followed by centrifugation at 4000 rpm for 15 min. For HSV infection and production assay, pre-plated 293T cells were infected with HSV (MOI = 0.02) after transfected with pCAGGS-hSLFN13 and related mutants for 2 h. The supernatants were collected 18 h after infection and diluted to infect pre-plated Vero cells for plaque formation and HSV titre determination.

For Zika virus production, albopictus C6/36 cells were infected with wide-type strain MR766 at 28 °C and virus supernatants were collected 5 days after infection. For Zika infection and production assay, A549 cells overexpressing pCAGGS-hSLFN13 and corresponding mutants were rinsed once with PBS, and infected with virus for 2 h at 37 °C. 48 h after infection, the supernatants were collected and diluted to infect pre-plated Vero cells for plaque assay.

### Assay for HIV infection

HIV^VSV-G^ was prepared by transfecting 293T cells with pNL4-3-ΔEnv-EGFP and pCMV-VSV-G using Lipofectamine 2000 (Thermo Scientific). The cell culture medium was collected and cleared as described above. Cells to be tested were infected for 6 h. At 24 h post-infection, GFP expression was measured to determine the relative infection efficiencies.

### Determination of viral RNA level

Virion supernatants were lysed and homogenised with TRIzol Reagent (Thermo Scientific) following the manufacturer’s protocol. Viral protein and DNA were removed by centrifugation at 4 °C for 15 min at 12,000 × *g*, and vRNA was extracted from the upper aqueous phase and subsequently reverse transcribed using PrimeScript RT reagent Kit (TaKaRa). The concentrations of total vRNA and unspliced vRNA were individually determined by qPCR with CFX96 Real-Time System (Bio-Rad) using SYBR Premix ExTaq Kit (TaKaRa). Primers used in qPCR are listed in Supplementary Table 4.

### Data availability

The X-ray crystallographic coordinates and structure factor files for rSLFN13-N has been deposited in the Protein Data Bank (PDB) under accession number 5YD0. All other data generated or analysed during this study are included in this published article, and are available from the corresponding author upon reasonable request.

## Electronic supplementary material

Supplementary Information(DOCX 9440 kb)
