# Supplementary material for: Structure of Schlafen13 reveals a new class of tRNA/rRNA- targeting RNase engaged in translational control
Source: Nat Commun. 2018 Mar 21;9:1165. doi: 10.1038/s41467-018-03544-x (PMC5862951; doi:10.1038/s41467-018-03544-x)
Supplement: Supplementary file 1 — Supplementary Information(DOCX 9440 kb) [file 41467_2018_3544_MOESM1_ESM.pdf]

Supplementary Information for

Structure of Schlafen13 reveals a new class of tRNA/rRNA-targeting RNase engaged in translational control

Jin-Yu Yang[1], Xiang-Yu Deng[2], Yi-Sheng Li[3,4], Xian-Cai Ma[5], Jian-Xiong Feng[1], Bing Yu[1], Yang Chen[1,7], Yi-Ling Luo[1], Xi Wang[3,8], Mei-Ling Chen[1], Zhi-Xin Fang[1], Fu-Xiang Zheng[5], Yi-Ping Li[5], Qian Zhong[1], Tie-Bang Kang[1], Li-Bing Song[1], Rui-Hua Xu[1], Mu-Sheng Zeng[1], Wei Chen[4,6], Hui Zhang[5], Wei Xie[2], Song Gao[1]

Corresponding authors: Song Gao (gaosong@sysucc.org.cn) and Wei Xie (xiewei6@mail.sysu.edu.cn); Jin-Yu Yang and Xiang-Yu Deng contributed equally to this work.

1. State Key Laboratory of Oncology in South China, Collaborative Innovation Center for Cancer Medicine, Sun Yat-sen University Cancer Center, Guangzhou 510060, China

2. State Key Laboratory for Biocontrol, School of Life Sciences, Sun Yat-Sen University, Guangzhou, Guangdong 510006, China

3. Laboratory for Functional Genomics and Systems Biology, Berlin Institute for Medical Systems Biology, Berlin 13125, Germany

4. Department of Biology, Southern University of Science and Technology, Shenzhen, Guangdong, China

5. Key Laboratory of Tropical Disease Control of Ministry of Education, Institute of Human Virology, Zhongshan School of Medicine, Sun Yat-sen University, Guangzhou, Guangdong 510080, China

6. Medi-X Institute, SUSTech Academy for Advanced Interdisciplinary Studies, Southern University of Science and Technology, Shenzhen, Guangdong, China

7. Current address: Laboratory of Metabolism, Center for Cancer Research, National Cancer Institute, National Institute of Health, Bethesda, MD 20892, USA

8. Current address: Division of Theoretical Systems Biology, German Cancer Research Center, D-69120, Germany

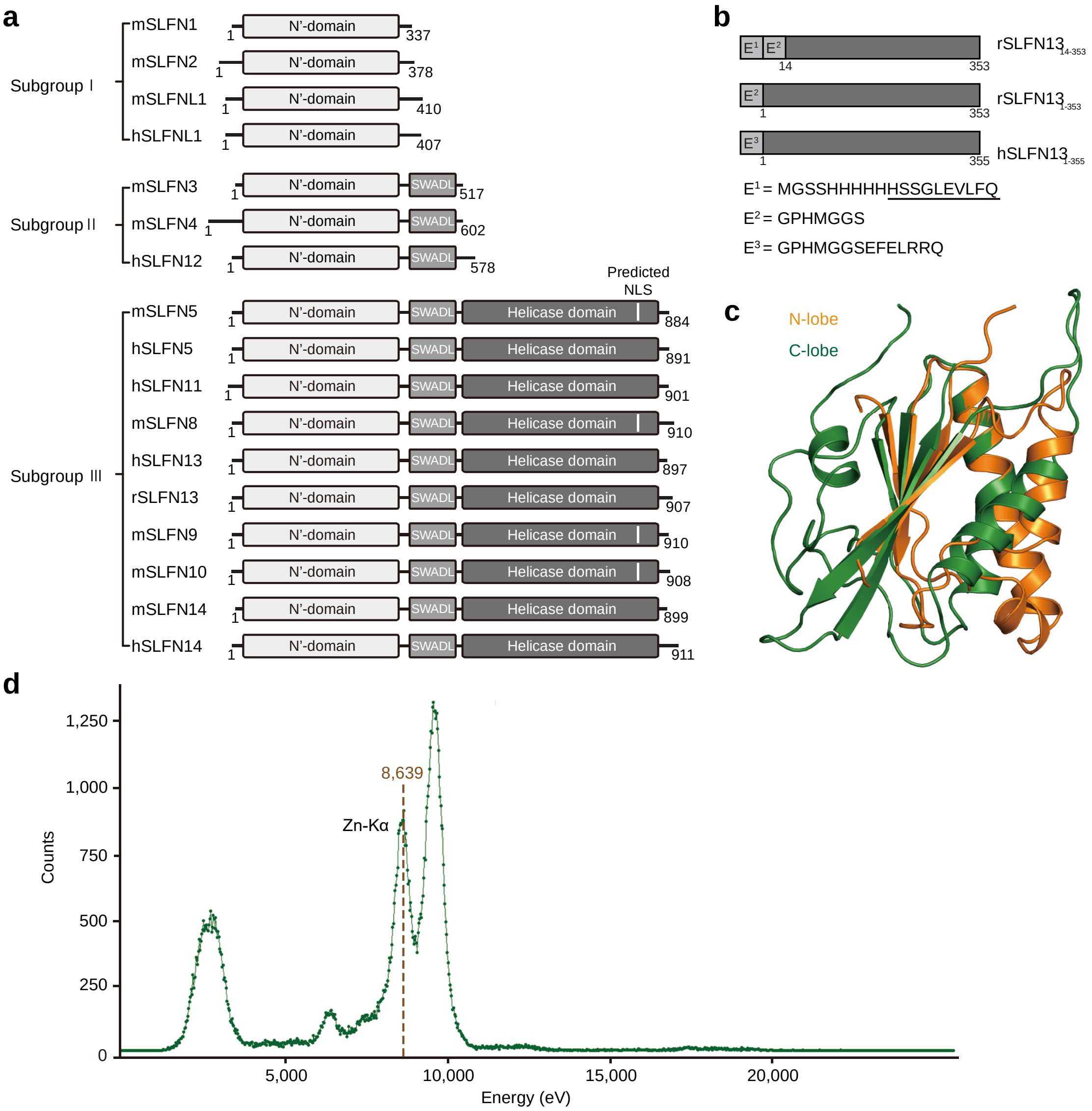

Supplementary Figure 1: Structural analysis of rSLFN13 N'-domain.

a Domain architecture of human and mouse SLFN proteins, as well as rat SLFN13, which are classified as three subgroups. SLFNL1: Schlafen-like protein 1. The predicted nuclear localization signal (NLS) for mouse SLFNs[1] is indicated by a white vertical bar.

b Schematic representation showing the composition of $rSLFN13_{14\text{-}353}$, $rSLFN13_{1\text{-}353}$ and $hSLFN13_{1\text{-}355}$ that were used in this study. E1, E2 and E3 denote the sequences of three N'-terminal extensions to corresponding SLFN constructs that are encoded by vector residues. Residues that are observable in the $rSLFN13_{14\text{-}353}$ structure are indicated by an underscore.

c Superposition of the N-lobe and C-lobe of $rSLFN13_{14\text{-}353}$ showing the consistent folding of the two subdomains.

d X-ray fluorescence scan over the $rSLFN13_{14\text{-}353}$ crystal. Emission of characteristic fluorescent X-ray at Zn-K edge confirmed the zinc incorporation in $rSLFN13_{14\text{-}353}$.

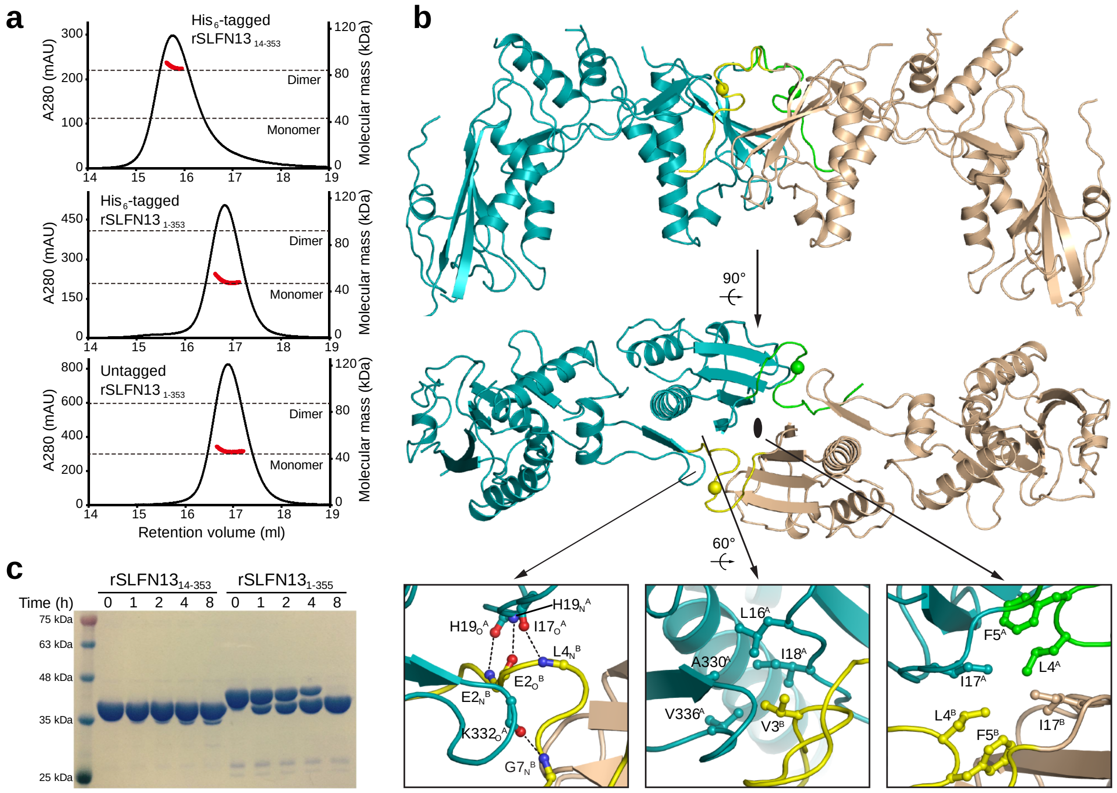

Supplementary Figure 2: Dimerization of rSLFN13$_{14\text{-}353}$.

a Dimerization properties of different rSLFN13-N constructs were assayed in analytical gel filtration coupled to RALS. Calculated molecular mass of each sample at the absorption peak of 280 nm are plotted in red. mAU, milli-absorption units.

b The putative dimeric interface of rSLFN13$_{14\text{-}353}$ in the crystal structure. The two monomers are coloured teal and wheat, and their vector-coded N'-terminal extensions are specified in green and yellow, respectively. The cleavage sites of the PreScission protease are indicated by spheres. Residues involved in the dimerization interface are shown as ball-and-stick models.

c Digestion test of different rSLFN13-N constructs. 0.72 µg PreScission protease was incubated with 240 µg rSLFN13$_{1\text{-}353}$ or rSLFN13$_{14\text{-}353}$.

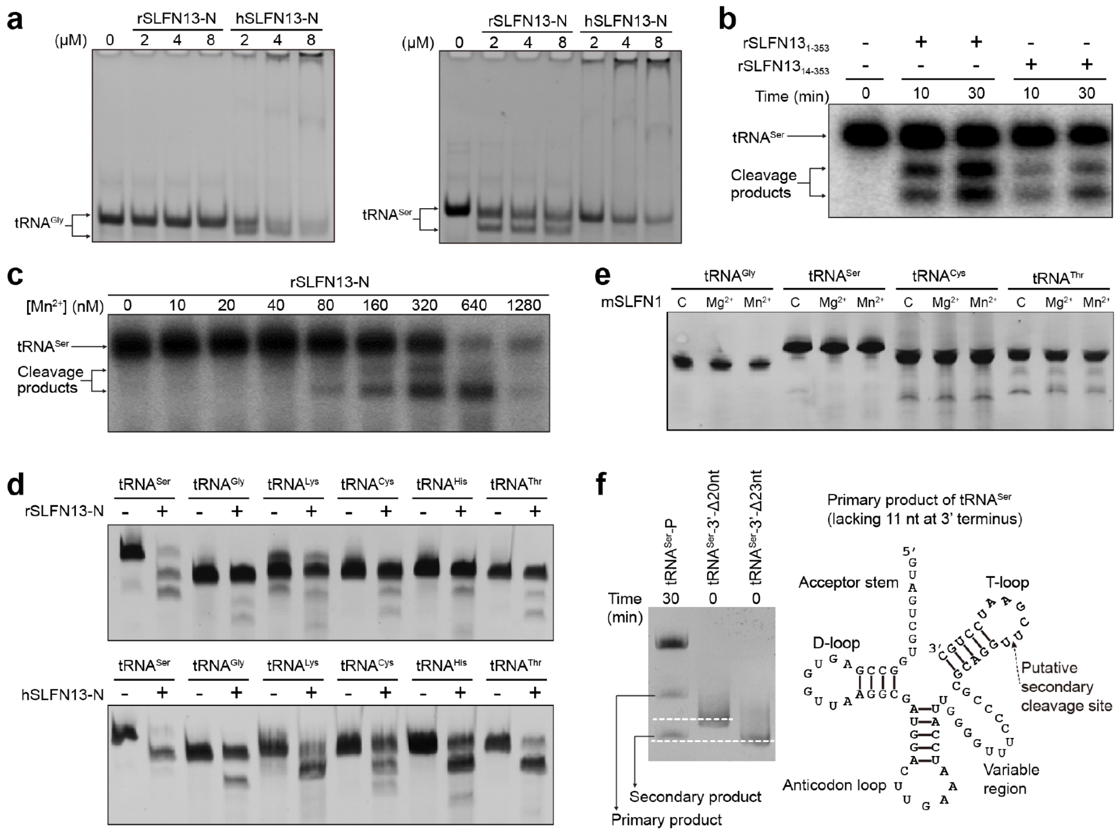

Supplementary Figure 3: Nucleolytic activity of SLFN13-N on tRNAs.

a tRNA degradation is found in EMSA in the presence of SLFN13-N.

b Both rSLFN13-N constructs can digest $tRNA^{Ser}$ in the presence of Mg^{2+}.

c Mn^{2+} prominently stimulates the nucleolytic activity of SLFN13-N. Mn^{2+} of increasing concentration was supplied to the cleavage reaction.

d Cleavage assays for rSLFN13-N and hSLFN13-N on different types of in *vitro* transcribed human tRNAs.

e mSLFN1 shows no tRNA cleavage activity in the presence of Mg^{2+} or Mn^{2+}.

f tRNA cleavage pattern of rSLFN13-N. The secondary cleavage products from $tRNA^{Ser}$ by $rSLFN13_{1\text{-}353}$ were compared with in *vitro* transcribed $tRNA^{Ser}$ that lacks 3'-terminal 20 nt ($tRNA^{Ser}$-3'-Δ20nt) or 23 nt ($tRNA^{Ser}$-3'-Δ23nt), and portrayed to the left. The migration of two truncated $tRNA^{Ser}$ markers is indicated by dashes lines. The sequence of human $tRNA^{Ser}$ is used here as representative.

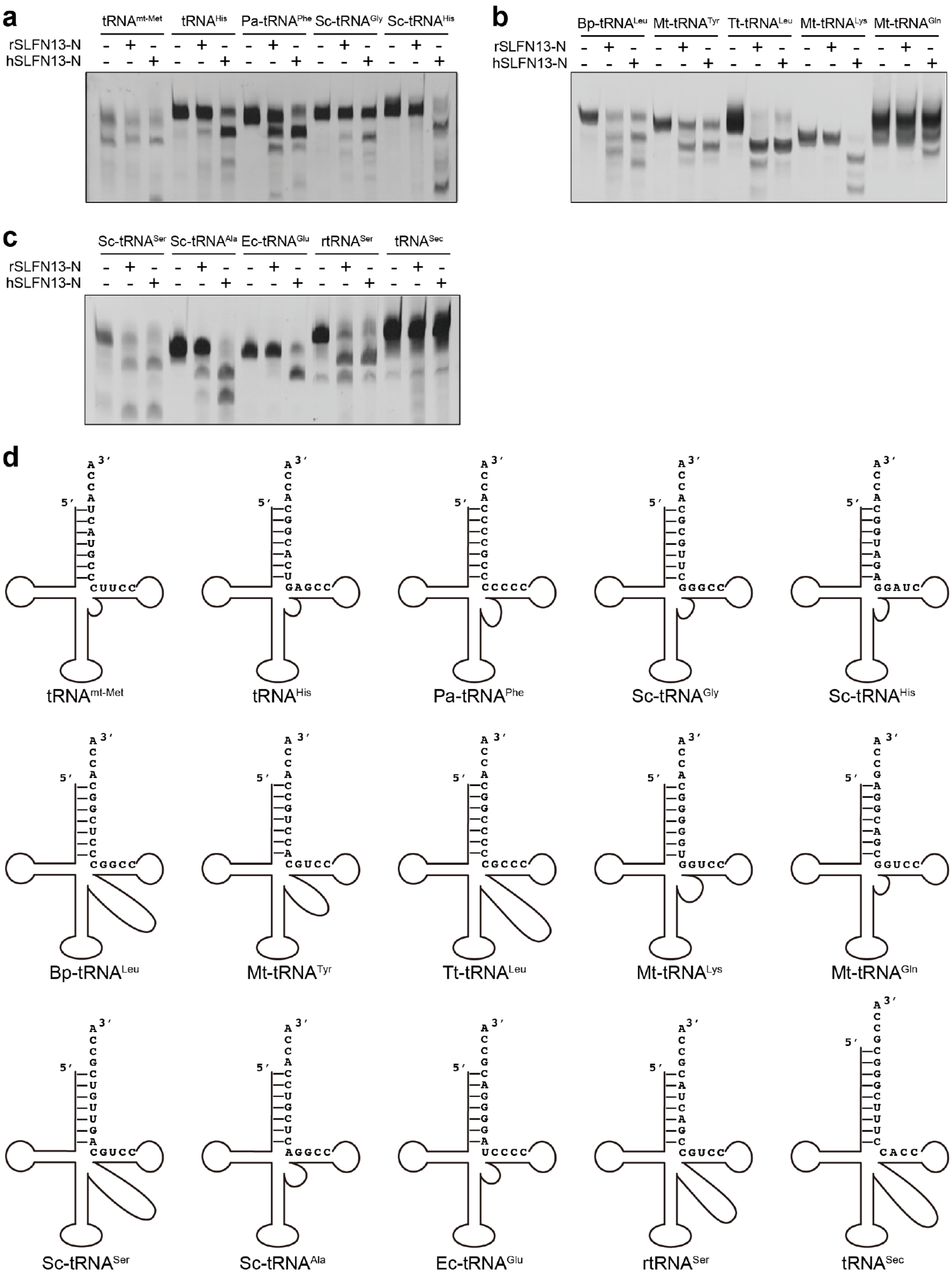

Supplementary Figure 4: Digestion activity of SLFN13 to other type tRNAs.

a-c Cleavage assays for rSLFN13-N and hSLFN13-N on different types of in *vitro* transcribed tRNAs in the presence of Mg^{2+}. tRNA substrates are: human $tRNA^{His}$, $tRNA^{Sec}$, mitochondrial $tRNA^{mt\text{-}Met}$ ($tRNA^{His}$, $tRNA^{Sec}$, $tRNA^{mt\text{-}Met}$), $tRNA^{Sec}$ denotes the transfer tRNA for selenium cysteine; *Pyrococcus abyssi* $tRNA^{Phe}$ (Pa-$tRNA^{Phe}$); *Saccharomyces cerevisiae* $tRNA^{Gly}$, $tRNA^{His}$, $tRNA^{Ser}$, $tRNA^{Ala}$ (Sc-$tRNA^{Gly}$, Sc-$tRNA^{His}$, Sc-$tRNA^{Ser}$, Sc-$tRNA^{Ala}$); *Bacillus paralicheniformis* $tRNA^{Leu}$ (Bp-$tRNA^{Leu}$); *Mycobacterium tuberculosi*s $tRNA^{Tyr}$, $tRNA^{Lys}$, $tRNA^{Gln}$ (Mt-$tRNA^{Tyr}$, Mt-$tRNA^{Lys}$, Mt-$tRNA^{Gln}$); *Thermus thermophiles* $tRNA^{Leu}$ (Tt-$tRNA^{Leu}$); *Escherichia coli* $tRNA^{Glu}$ (Ec-$tRNA^{Glu}$); rabbit $tRNA^{Ser}$ ($rtRNA^{Ser}$).

d Schematic drawing of different tRNAs used in **a-c**. Sequences from the 3'-terminus to the T-loop are specified. Paired bases are indicated. The variation loops are drawn according to their sizes.

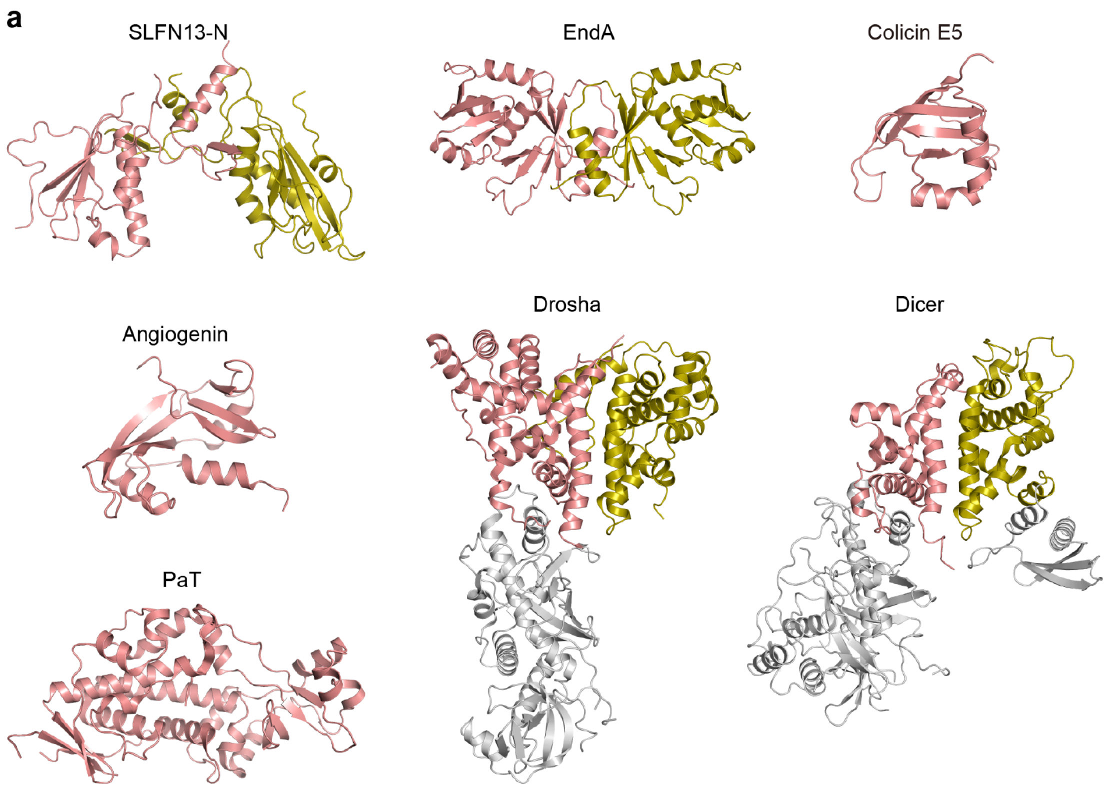

b

No:	Chain	Z	rmsd	lali	nres	%id	PDB	Description
1:	3lmm-D	9.0	5.4	123	556	13		MOLECULE: UNCHARACTERIZED PROTEIN;
2:	3lmm-C	8.9	5.6	127	477	13		MOLECULE: UNCHARACTERIZED PROTEIN;
3:	3lmm-B	8.9	5.4	129	555	14		MOLECULE: UNCHARACTERIZED PROTEIN;
4:	3lmm-A	8.7	5.5	128	492	14		MOLECULE: UNCHARACTERIZED PROTEIN;
5:	2kyy-A	8.0	2.4	105	153	10		MOLECULE: POSSIBLE ATP-DEPENDENT DNA HELICASE RECG-RELATED
6:	2vrt-D	6.4	4.3	99	487	11		MOLECULE: RIBONUCLEASE E;
7:	2vmk-A	6.4	3.4	102	489	11		MOLECULE: RIBONUCLEASE E;
8:	2vmk-C	6.4	6.2	99	474	10		MOLECULE: RIBONUCLEASE E;
9:	2bx2-L	6.0	3.2	104	500	11		MOLECULE: RIBONUCLEASE E;
10:	3evz-A	5.9	2.8	88	197	3		MOLECULE: METHYLTRANSFERASE;

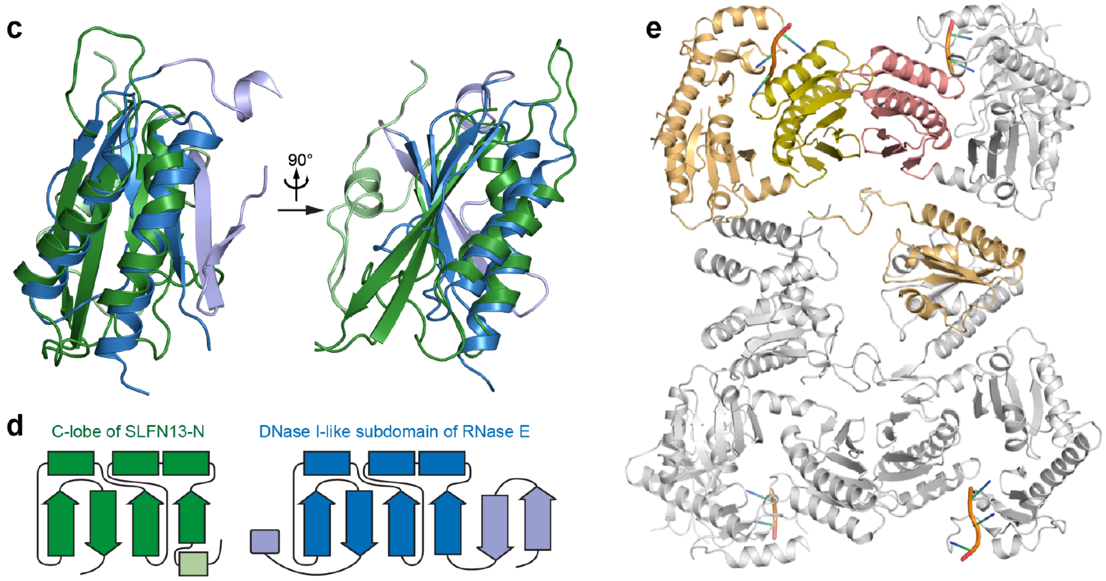

Supplementary Figure 5: Structural comparison of rSLFN13-N with other RNases.

a Structures of rSLFN13-N and other endonucleases, namely tRNA-targeting EndA (PDB ID 1A79), colicin E5 (2A8K), PaT (4O87), angiogenin (1AGI), dsRNA-targeting Drosha (5B16)

and Dicer (2FFL), are shown as ribbon-typed models. For rSLFN13-N, EndA, Drosha and Dicer, the dimeric or pseudodimeric regions that possess the nucleolytic activity are coloured salmon and olive, and the rest portions of Drosha and Dicer are coloured grey. Colicin E5, PaT and angiogenin are coloured salmon.

b Top 10 hits of the existing structures homologous to rSLFN13-N, output from the Dali server.

c Structural superposition of rSLFN13-N C-lobe and the DNase I-like subdomain of RNase E (2BX2). Cα atoms of 78 residues are aligned with a rmsd of 3.14 Å. Colour as in **Fig. 3a**.

d Comparison of the structural topology between rSLFN13-N C-lobe and the DNase I-like subdomain of RNase E.

e The structure of RNase E catalytic domain tetramer in complex with RNA (2VRT). A dimerized DNase I-like subdomain pair is coloured either salmon or olive for each protomer. One chain is coloured light orange, and other portions of the tetramer are coloured grey.

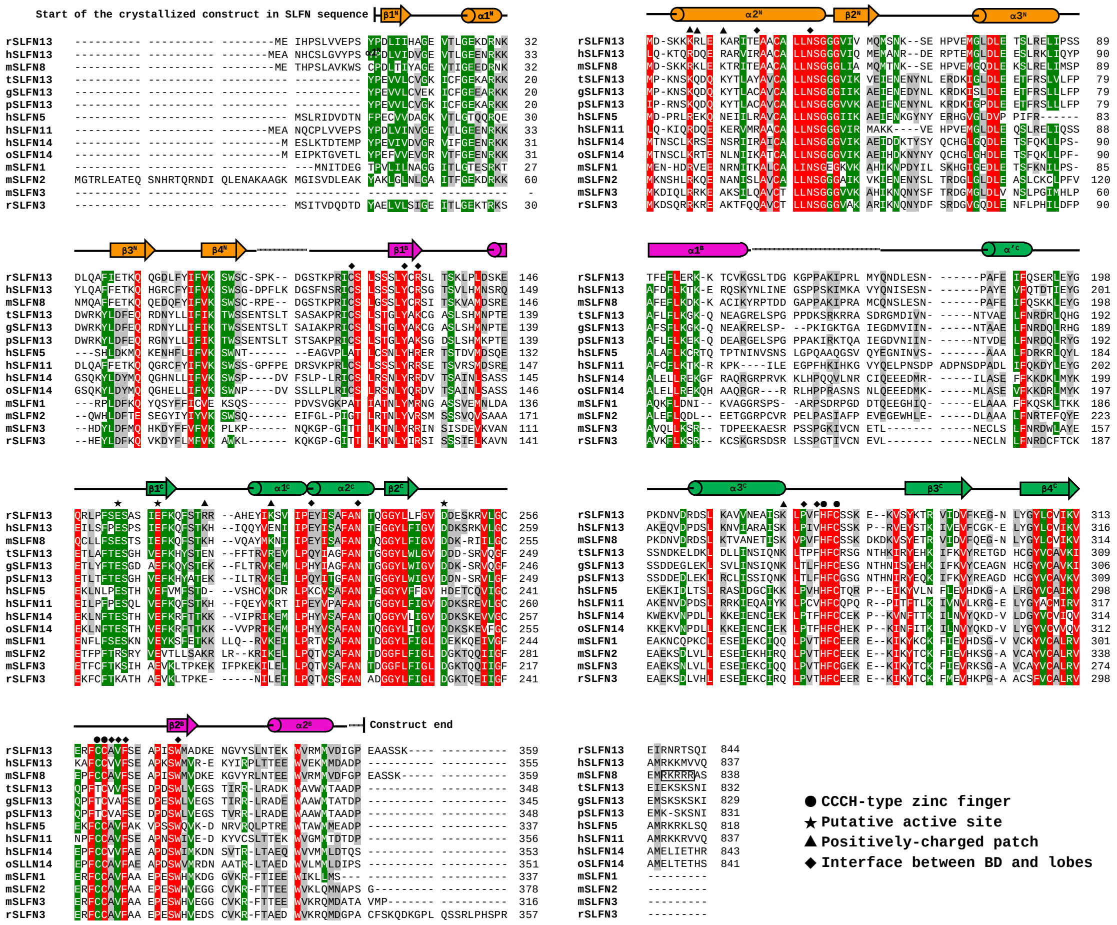

Supplementary Figure 6: Sequence alignment of SLFN family within the N'-domain.

Amino acid sequences of rat (r)SLFN13 (UniProt accession Q5U311), human (h)SLFN13 (Q68D06), mouse (m)SLFN8 (Q7TMF1), *Tyto alba* (t)SLFN13 (A0A093HQW8), *Gavia stellata* (g)SLFN13 (A0A093GYA7), *Pygoscelis adeliae* (p)SLFN13 (A0A093P2N6), hSLFN5 (Q08AF3), hSLFN11 (Q7Z7L1), hSLFN14 (P0C7P3), *Oryctolagus cuniculus* or rabbit (o)SLFN14 (G1SRW8), mSLFN1 (Q9Z0I7), mSLFN2 (Q9Z0I6), mSLFN3 (Q9Z0I5) and rSLFN3 (Q9TPX1) are aligned using Clustal W[2]. Residues with a conservation of 100% are in red shades, greater than 80% in green shades and 60% in grey shades, respectively. α-helices are shown as cylinders and β-strands as arrows for rSLFN13 above the sequences. The secondary structure signs are coloured and labelled as in **Fig. 1c**. Regions not resolved in the crystal structure are indicated by dashed lines. Important residues that are discussed in the paper are specified by different symbols according to their structural and functional roles. mSLFN8 was predicted to carry an NLS[1]. The sequences of the selected SLFNs are aligned at the NLS of mSLFN8 which is indicated by a black box. Sequences at this region are not very conserved, and no apparent NLS is found for rSLFN13 or hSLFN13.

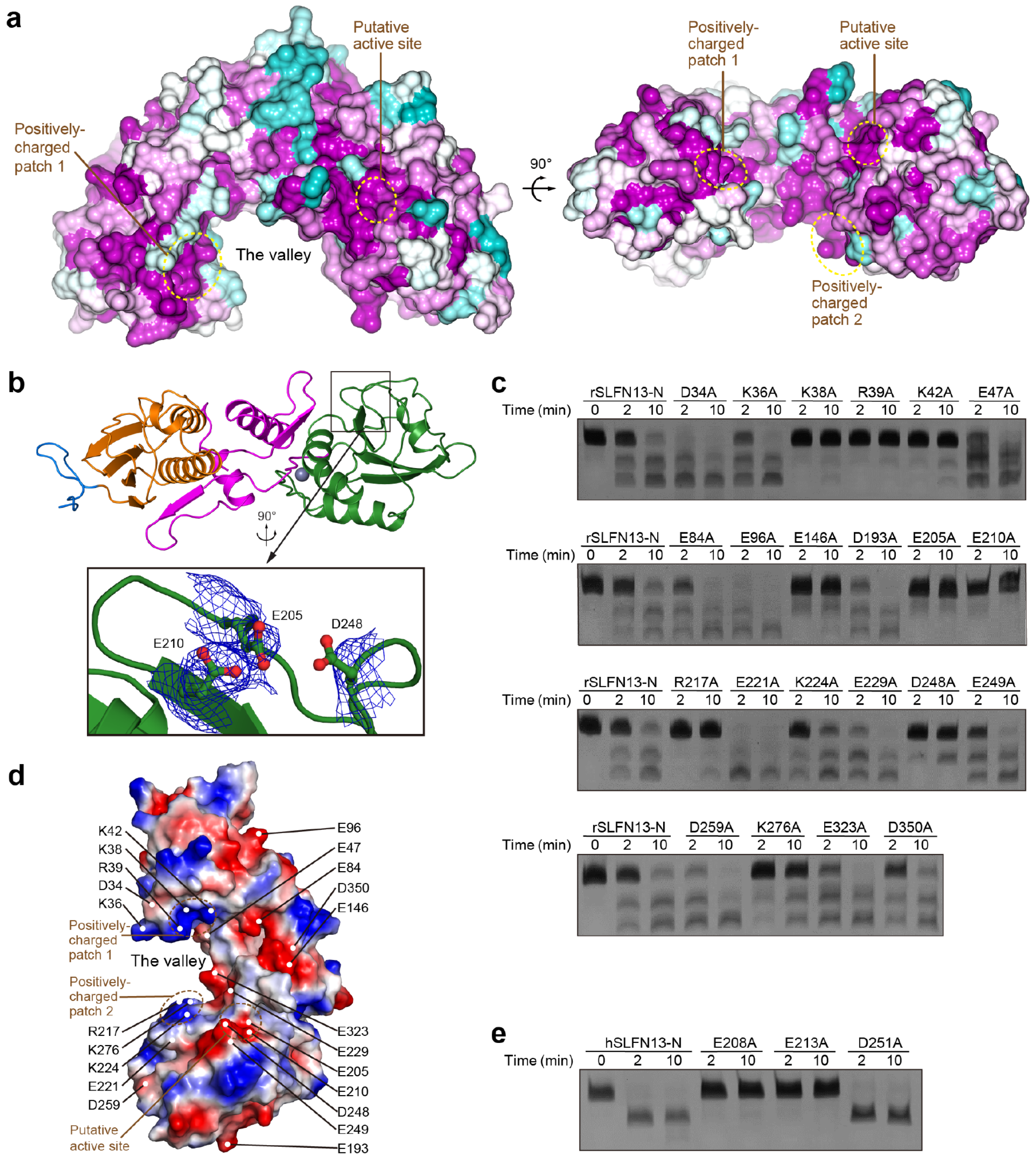

Supplementary Figure 7: The tRNA manipulation model of SLFN13-N.

a Surface conservation plot of rSLFN13-N. The plot was generated using the ConSurf Server[3] based on the sequence alignment shown in **Supplementary Fig. 6**, and the crystal structure of rSLFN13$_{14\text{-}353}$. Residues are coloured from magenta (highly-conserved) to cyan (non-conserved).

b The electron density of the putative active site. The density is shown as blue mesh at a contour level of 1.0 σ. Putative catalytic residues are shown as ball-and-stick models.

c tRNA cleavage assay for wild-type rSLFN13-N and corresponding mutants in the presence of Mg^{2+}. 1 μM protein was incubated with 500 nM $tRNA^{Ser}$.

d The electrostatic surface potentials of rSLFN13$_{14\text{-}353}$, coloured and depicted as **Fig. 3b**. Positions of the residues tested in **c** are specified.

e tRNA cleavage assay for wild-type hSLFN13-N and mutants of putative catalytic residues. 1 μM protein was incubated with 500 nM $tRNA^{Ser}$.

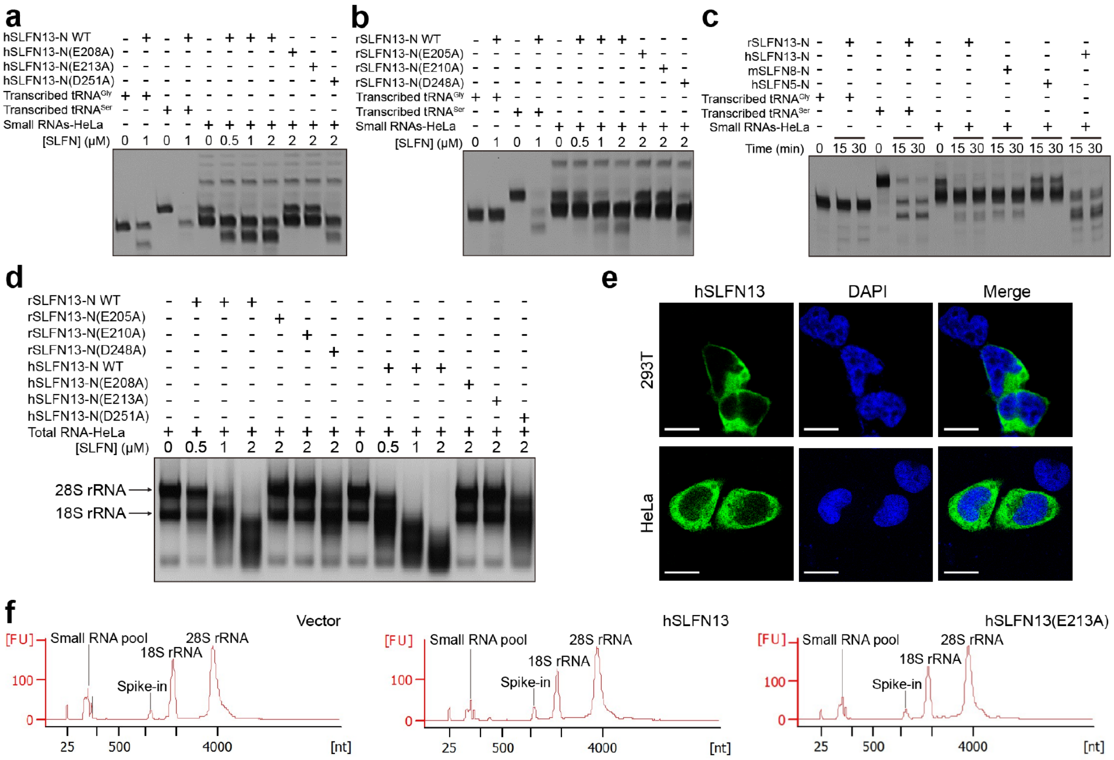

Supplementary Figure 8: SLFN13-N can digest native tRNAs.

a Cleavage assay for hSLFN13-N or corresponding mutants in the presence of Mg^{2+} on total small RNAs extracted from HeLa cells. These small RNAs are mainly composed of tRNAs.

b Cleavage assays for rSLFN13-N and corresponding mutants on total small RNAs extracted from HeLa cells.

c Cleavage assays for the N'-domains of different SLFN proteins on total small RNAs extracted from HeLa cells.

d Cleavage assay on native rRNAs. 4 µg of total RNA extracted from HeLa cells was used for each reaction.

e Subcellular localization of hSLFN13 tested by fluorescence assay. Scale bars, 10 µm.

f Quantification of cellular RNA by Bioanalyzer. The small RNA pool corresponds to RNAs with length under 200 nt, which are mainly composed of tRNAs, 5S rRNA and 5.8S rRNA. The areas of the small RNA pool, 18S rRNA and 28S rRNA peaks were individually integrated and normalized with the area of corresponding spike-in RNA.

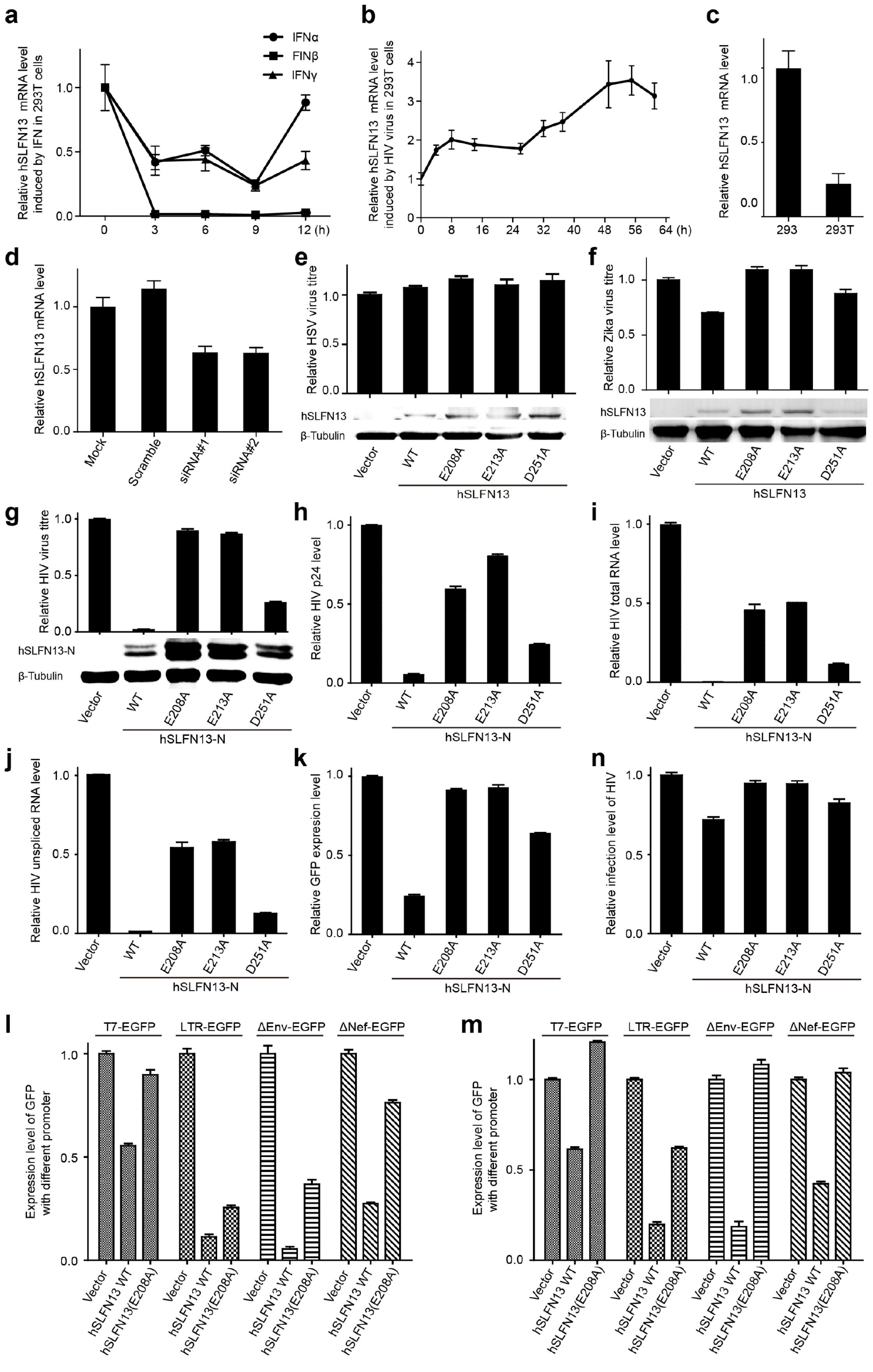

Supplementary Figure 9: The antiviral activity of hSLFN13.

a IFNs did not induce hSLFN13 expression in 293T cells. Error bar indicates s.d. (n = 3).

b The infection of pseudotyped HIV-1 (HIV^{VSV-G}) escalated hSLFN13 mRNA level in 293T cells.

c Comparison of endogenous mRNA levels of hSLFN13 in 293 and 293T cells.

d Knock-down of hSLFN13 expression in 293 cells by siRNAs. mRNA levels of hSLFN13 were measured by qPCR.

e Production of HSV Virus was assayed by titrated infection and plaque assay. Expression of hSLFN13 WT and mutants was confirmed by Western blot.

f Production of Zika Virus was assayed by titrated infection and plaque assay. Expression of hSLFN13 WT and mutants was confirmed by Western blot.

g Production of HIV Virus was assayed by titrated infection and the GFP level. Expression of hSLFN13-N WT and mutants was confirmed by Western blot.

h Viral particle content in supernatants was analyzed by p24 ELISA.

i, **j** Extracellular vRNA concentration was determined by qPCR of total vRNA (**i)** and unspliced vRNA (**j)**.

k hSLFN13-N suppressed GFP production from the viral plasmids.

l, **m** Suppression of GFP production from plasmids with T7 promoter (T7-EGFP) or HIV promoter (LTR-EGFP, ΔEnv-EGFP and ΔNef-EGFP) by hSLFN13 (**l)** and hSLFN13-N (**m)**, as well as corresponding mutants. LTR-EGFP: EGFP with long terminal repeat promoter; ΔEnv-EGFP: pNL4-3-ΔEnv-EGFP; ΔNef-EGFP: pNL4-3-ΔNef-EGFP.

n Examination for the infection efficiency of HIV^{VSV-G} in cells pre-transfected with hSLFN13-N WT or mutants.

Supplementary Table 1: Sequences of the tRNA transcripts

tRNA	Sequences
$tRNA^{Ser}$	5’ –GUAGUCGUGGCCGAGUGGUUAAGGCGAUGGACUUGAAAUCCAUUG<br>GGGUUUCCCCGCGCAGGUUCGAAUCCUGCCGACUACGCCA–3’
$tRNA^{Gly}$	5’ –GCGCCGCUGGUGUAGUGGUAUCAUGCAAGAUUCCCAUUCUUGCGA<br>CCCGGGUUCGAUUCCCGGGCGGCGCACCA–3’
$rtRNA^{Ser}$	5’ –GUAGUCGUGGCCGAGUGGUUAAGGCGAUGGACUUGAAAUCCAUUG<br>GGGUCUCCCCGCGCAGGUUCGAAUCCUGCCGACUACGCCA–3’
$tRNA^{Lys}$	5’ -GCCCGGAUAGCUCAGUCGGUAGAGCAUCAGACUUUUAAUCUGAGG<br>GUCCAGGGUUCAAGUCCCUGUUCGGGCGCCA-3’
$tRNA^{Cys}$	5’ -GGGGGUAUAGCUCAGGGGUAGAGCAUUUGACUGCAGAUCAAGAG<br>GUCCCUGGUUCAAAUCCAGGUGCCCCCUCCA-3’
$tRNA^{His}$	5’ -GGCCGUGAUCGUAUAGUGGUUAGUACUCUGCGUUGUGGCCGCAGC<br>AACCUCGGUUCGAAUCCGAGUCACGGCACCA-3’
$tRNA^{Thr}$	5’ -GGCUCCAUAGCUCAGGGGUUAGAGCACUGGUCUUGUAAACCAGGG<br>GUCGCGAGUUCAAAUCUCGCUGGGGCCUCCA-3’
$tRNA^{mt\text{-}Met}$	5’ -AGUAAGGUCAGCUAAAUAAGCUAUCGGGCCCAUACCCCGAAAAUGU<br>UGGUUAUACCCUUCCCGUACUACCA-3’
Pa-$tRNA^{Phe}$	5’ –GGGGCGGUAGCUCAGCCUGGGAGAGCACCGGACUGAAGAUCCGGGU<br>GUCGGGGGUUCAAAUCCCCCCCGCCCCACCA-3’
Sc-$tRNA^{Gly}$	5’ –GCGCAAGUGGUUUAGUGGUAAAAUCCAACGUUGCCAUCGUUGGGCC<br>CCCGGUUCGAUUCCGGGCUUGCGCACCA-3’
Sc-$tRNA^{His}$	5’ –GGCCAUCUUAGUAUAGUGGUUAGUACACAUCGUUGUGGCCGAUGAA<br>ACCCUGGUUCGAUUCUAGGAGAUGGCACCA-3’
Bp-$tRNA^{Leu}$	5’ –GCCGGGGUGGUGGAAUUGGCAGACACACAGGACUUAAAAUCCUGCG<br>GUAGGUGACUACCGUGCCGGUUCAAGUCCGGCCCUCGGCACC-3’
Mt-$tRNA^{Tyr}$	5’ –GGCAGGUUGCCCGAGCGGCCAAUGGGAGCGGACUGUAAAUCCGUCG<br>CGAAAGCUACGCAGGUUCGAAUCCUGCACCUGCCACCA-3’
Tt-$tRNA^{Leu}$	5’ –GCCGGGGUGGCGGAACCGGUAGACGCGGCAGACUCAAAAUCUGCUG<br>UCCGCAAGGACGUGCGGGUUCGAGUCCCGCCCCCGGCACCA-3’
Mt-$tRNA^{Lys}$	5’ –GGCCCCUAUAGCUCAGUUGGUAGAGCUACGGACUUUUAAUCCGCAG<br>GUCCCAGGUUCGAGUCCUGGUGGGGGCACCA-3’
Mt-$tRNA^{Gln}$	5’ –TCCGTCGTGGTGTAATCGGCAGCACCTCTGATTTTGGTTCAGATAG<br>TTCAGGTTCGAGTCCTGGCGACGGAGCCA-3’
Sc-$tRNA^{Ser}$	5’ –GGCAACUUGGCCGAGUGGUUAAGGCGAAAGAUUAGAAAUCUUUUGG<br>GCUUUGCCCGCGCAGGUUCGAGUCCUGCAGUUGUCGCCA-3’
Sc-$tRNA^{Ala}$	5’ –GGGCGUGUGGCGUAGUCGGUAGCGCGCUCCCUUAGCAUGGGAGAGG<br>UCUCCGGUUCGAUUCCGGACUCGUCCACCA-3’
Ec-$tRNA^{Glu}$	5’ –GUCCCCUUCGUCUAGAGGCCCAGGACACCGCCCUUUCACGGCGGUA<br>ACAGGGGUUCGAAUCCCCUAGGGGACGCCA-3’
$tRNA^{Sec}$	5’ –GCCCGGAUGAUCCUCAGUGGUCUGGGGUGCAGGCUUCAAACCUGUA<br>GCUGUCUAGCGACAGAGUGGUUCAAUUCCACCUUUCGGGCGCCA-3’

Supplementary Table 2: Sequences of the nucleic acids substrates

Nucleic acids	Sequences
dsDNA	Sense: 5'–GGCTTTTGACCTTTATGC–3'<br>Anti-sense: 5'–GCATAAAGGTCAAAAGCC–3'
ssDNA	5'–GGCTTTTGACCTTTATGC–3'
dsRNA	Sense: 5'–GGCUUUUGACCUUUAUGC–3'<br>Anti-sense: 5'–GCAUAAAGGUCAAAAGCC–3'
ssRNA	5'–GGCUUUUGACCUUUAUGC–3'
DNA/RNA duplex	Sense: 5'–GGCTTTTGACCTTTATGC–3'<br>Anti-sense: 5'–GCAUAAAGGUCAAAAGCC–3'
Stem-loop RNA	5'–AUAAAGGUCAUUCGCAAGAGUGGCCUUUAU–3'

Supplementary Table 3: tRNA and rRNA probes for Northern blot

Probe	Sequences
$tRNA^{Ser}$	5'–AACCACTCGGCCACCTCGTC–3'
$tRNA^{Gly}$	5'–TACCACTGAACCACCAATGC–3'
$tRNA^{Lys}$	5'–ACCGACTGAGCTAGCCGGGC–3'
5S rRNA	5'–AAGCCTACAGCACCCGGTAT–3'

Supplementary Table 4: Sequences of qPCR primers

Primer	Sequences
Total vRNA	Forward: 5'–CTGGCTAACTAGGGAACCCACTGCT–3'<br>Reverse: 5'–GCTTCAGCAAGCCGAGTCCTGCGTC–3'
Unspliced vRNA	Forward: 5'–ATAATCCACCTATCCCAGTAGGAGAAA–3'<br>Reverse: 5'–TTTGGTCCTTGTCTTATGTCCAGAATGC–3'
hSLFN13	Forward: 5'– GAGAAAATGATGGACGCAGAT–3'<br>Reverse: 5'– AGACTCAAAGGCCTCAGCAA–3'
β-actin	Forward: 5'– GTGAAGGTGACAGCAGTCGGT–3'<br>Reverse: 5'– AAGTGGGGTGGCTTTTAGGAT–3'

Supplementary References

1. Neumann, B., Zhao, L., Murphy, K. & Gonda, T. J. Subcellular localization of the Schlafen protein family. *Biochem. Biophys. Res. Commun.* 370, 62–66 (2008).

2. Thompson, J. D., Higgins, D. G. & Gibson, T. J. CLUSTAL W: improving the sensitivity of progressive multiple sequence alignment through sequence weighting, position-specific gap penalties and weight matrix choice. *Nucleic Acids Res.* 22, 4673–4680 (1994).

3. Landau, M. et al. ConSurf 2005: the projection of evolutionary conservation scores of residues on protein structures. *Nucleic Acids Res.* 33, W299–W302 (2005).
